# Recruitment of apolipoprotein E facilitates Herpes simplex virus 1 attachment and release

**DOI:** 10.1038/s44298-025-00099-9

**Published:** 2025-02-22

**Authors:** Lifeng Liu, Fouzia Bano, Dario Valter Conca, Konrad Thorsteinsson, Sanduni Wasana Jayaweera, Damien Avinens, Hudson Pace, Hugo Lövheim, Anders Olofsson, Marta Bally

**Affiliations:** 1 Department of Clinical Microbiology, Umeå University, Umeå, Sweden; 2 Wallenberg Centre for Molecular Medicine, Umeå University, Umeå, Sweden; 3 Umeå Centre for Microbial Research, Umeå University, Umeå, Sweden; 4 Department of Community Medicine and Rehabilitation, Umeå University, Umeå, Sweden

**Keywords:** Microbiology, Virology, Herpes virus

## Abstract

Human apolipoprotein E (ApoE) has been shown to play important roles during primary infection and pathogenesis of several viruses. Furthermore, epidemiological studies suggest that interactions between ApoE 4 and herpes simplex virus type-1 (HSV1) could associate with higher risk of Alzheimer’s disease. Nevertheless, little is known about the ApoE-HSV1 interactions at molecular levels. Here, we investigate the effects of ApoE on HSV1 infection in vitro. Our results show that ApoE promotes HSV1 growth, which is attributed to the incorporation of ApoE into HSV1 particles. Using both biological and biophysical approaches, we conclude that ApoE-coated HSV1 demonstrates a more efficient attachment to and faster release from the cell surface. Mechanistic studies reveal that ApoE modifies HSV1 interactions with heparan sulfate, thereby modulating interactions between HSV1 and the cell surface. Overall, our results provide new insights into the roles of ApoE during HSV1 infections which may inspire future studies on Alzheimer’s disease etiology.

## Introduction

Apolipoprotein E (ApoE) is an important human protein, primarily known for its functions in lipid metabolism. Yet, recent investigations reveal that its biological role spans beyond its conventional function in cholesterol transport; the protein has been shown to play roles in immune regulation, in the development of cardiovascular and neurological diseases, and in inflammation^1^. In the context of virus infection, ApoE has been suggested to influence infection by several viral pathogens, including, hepatitis B virus (HBV), hepatitis C virus (HCV), human immunodeficient virus (HIV), and herpes simplex virus type-1 (HSV1)^2^. Additionally, ApoE allele 4, *APOE 4*, is a recognized important risk factor for Alzheimer’s disease development^3^. In this context, population-based cohort studies have suggested that the risk factor for the disease is increased when associated with herpes simplex virus 1 (HSV1) infection^4–11^, although an association between HSV1 with Alzheimer’s diseases remains a matter of debate in the scientific community^12–16^. Despite the suggested involvements of ApoE in the infection process of HSV1, little is known about how both interact mechanistically on a molecular level.

ApoE is a polymorphic and multi-functional protein, 299 amino acids in length and containing two structural domains identified as the N-terminal (amino acids 1–191) and C-terminal domains (amino acids ∼206–299), connected by a linker. Functional regions located in the two domains include the receptor binding and heparan sulfate (HS) binding regions in the N-terminal domain^17^, as well as the lipid binding and self-association regions in the C-terminal domain (reviewed in^1^). The *ApoE* gene is located on human chromatin 19^18^ and produces three major alleles, that encode three corresponding protein products termed ApoE 2, ApoE 3, and ApoE 4. These three ApoE isoforms differ at only two amino acid residues: 112 and 158; ApoE 3 has a cysteine at residue 112 and an arginine at 158; while at both sites ApoE 2 has cysteines and ApoE 4 has arginines. These single amino acid substitutions lead to important changes in the ApoE structure, which in turn results in differences in the characteristics of the protein’s receptor, lipid, and HS-binding abilities (reviewed in ref. ^19^). For example, ApoE 2 shows significantly lower binding affinity than ApoE 3 or 4 to the low-density lipoprotein (LDL) receptor on cultured cells, which is thought to be a result of the missing arginine at 158^20^. The arginine 112 in ApoE 4 enables unique domain interactions, accounting for its preference for very low-density lipoproteins (VLDL), in comparison to binding to high-density lipoprotein by ApoE 2 or ApoE 3^21^. In addition, a biomolecular interaction study using surface plasmon resonance has observed that ApoE 4 shows significantly higher binding than ApoE 3 or ApoE 2 to the glycosaminoglycans (GAG) heparan sulfate and dermatan sulfate found on the cell surface, which is postulated to be a result of electrostatic interactions with the N-terminal domain^22^. For decades, although carrying the allele *ApoE 4* has been seen as the most important risk factor for the development of sporadic Alzheimer’s disease (see review^3^), mechanistic insights on why ApoE 4 increases Alzheimer’s disease susceptibility are widely lacking, and the protein alone is not a strong risk factor for Alzheimers’s disease development, suggesting the involvement of other factors, such as an infection history with some human neurotropic pathogens. Some studies have suggested that herpesvirus infection, and in particular infection with HSV1, could be one of the important pathogenic factors for Alzheimer’s disease development^23^. However, this hypothesis remains a matter of debate^24^ with some studies strengthening this hypothesis^12–14^, while other studies have reported negligible association between HSV1 and Alzheimer's disease^15,16^.

HSV is a human neurotropic virus member of the alpha Herpesviridae subfamily, which exists as two major serotypes HSV1 and HSV2. The prevalence of infection is estimated to be as high as 67% for HSV1 and 13% for HSV2 among people under age 50 (“Herpes simplex virus”. World Health Organization. 31 January 2017). One of the biological features of HSV infection is that the virus can produce lytic or latent infections, depending on the target cell types. A lytic lifecycle of HSV1 occurs at its primary infection site, oral epithelial layers, from which the infection can spread to the trigeminal ganglion neurons where HSV1 latency is established^25^. The HSV virion is composed of three structural parts: an icosahedral capsid enclosing a linear, double-stranded DNA viral genome; a layer of proteins called tegument surrounding the capsid shell, and a glycoprotein-containing lipid membrane envelope as the outermost layer, wrapping both the tegument and nucleocapsid. The HSV DNA genome, ~152 kb pairs in length, encodes about 80 viral proteins, among which at least 12 of them are glycoproteins displayed on the envelope. Consistent with the large variety of glycoproteins found in the virion, HSV1 entry is a complex and versatile process which depends on the infected tissue and on the infection circumstances. Prior to entry, virus attachment is mediated by the interactions between virus glycoprotein B and C and GAGs on the cell surface, HS^26^ or chondroitin sulfate proteoglycans^27^. Recent studies have characterized interactions between HSV glycoprotein B or C and various GAGs^28–31^ and revealed that the interactions are important both for virus attachment and virus particle transport to entry receptors on the cell surface^32^. These studies also highlight that the characteristics of such interactions need to be tightly regulated, not only to ensure efficient virus recruitment during entry but also efficient virus release upon egress. Glycoprotein o-glycosylation^28,33,34^ but also increased heparinase expression upon infection^35,36^ have been proposed as important factors contributing to a balanced GAG-virus interaction at the cell surface.

Recent studies have revealed that ApoE plays important roles during viral infections and pathogenesis^1,2^. ApoE expression is upregulated in HIV-infected macrophages but inhibits HIV infection when overexpressed in the 293 T cell line^37^. In contrast, hepatotropic virus hepatitis B (HBV) and hepatitis C (HCV) viruses require ApoE for efficient infection and virus production, and ApoE has also been co-purified together with HBV and HCV particles, suggesting incorporation of the protein into virus particles^38–42^. Mechanistic studies suggest that virus attachment to heparan sulfate proteoglycan (HSPG) on the cell surface is facilitated by ApoE for both HBV and HCV^43,44^ and that virus assembly or egress of HCV also benefits from ApoE^45–47^. However, none of these effects are isoform-dependent. Isoform-dependent effects have been observed in some HSV1 studies. Transgenic mice studies show that in comparison to ApoE 3, ApoE 4 leads to more brain access^48^ and latency to HSV1^49,50^, as well as to more severe pathogenesis^51^. Moreover, ApoE-derived peptides were found to inhibit HSV1 infection^52–54^, likely by targeting HSPGs, a shared cell-surface receptor for both ApoE and HSV1^26,55^. On the other hand, proviral effects of ApoE have been suggested in ApoE knockout mice, where HSV1 infection and transmission is decreased in different organs, such as the spinal cord and brain^56^. Despite pathogenesis studies in animal models, and although similar studies have been done with other viruses, such as HIV, HBV, or HCV, little is known about ApoE effects on HSV1 growth at molecular levels. Here we investigate HSV1 infection on cultured cell lines in the presence of different ApoE isoforms. Our results reveal that the association of ApoE with HSV1 particles promotes infection by facilitating both virus attachment to and release from the cell surface, and that this is the result of a modified interaction between ApoE-carrying virus particles and heparan sulfate on the cell surface.

## Results

### HSV1 infection is accelerated by the presence of ApoE

**Fig. 1:**
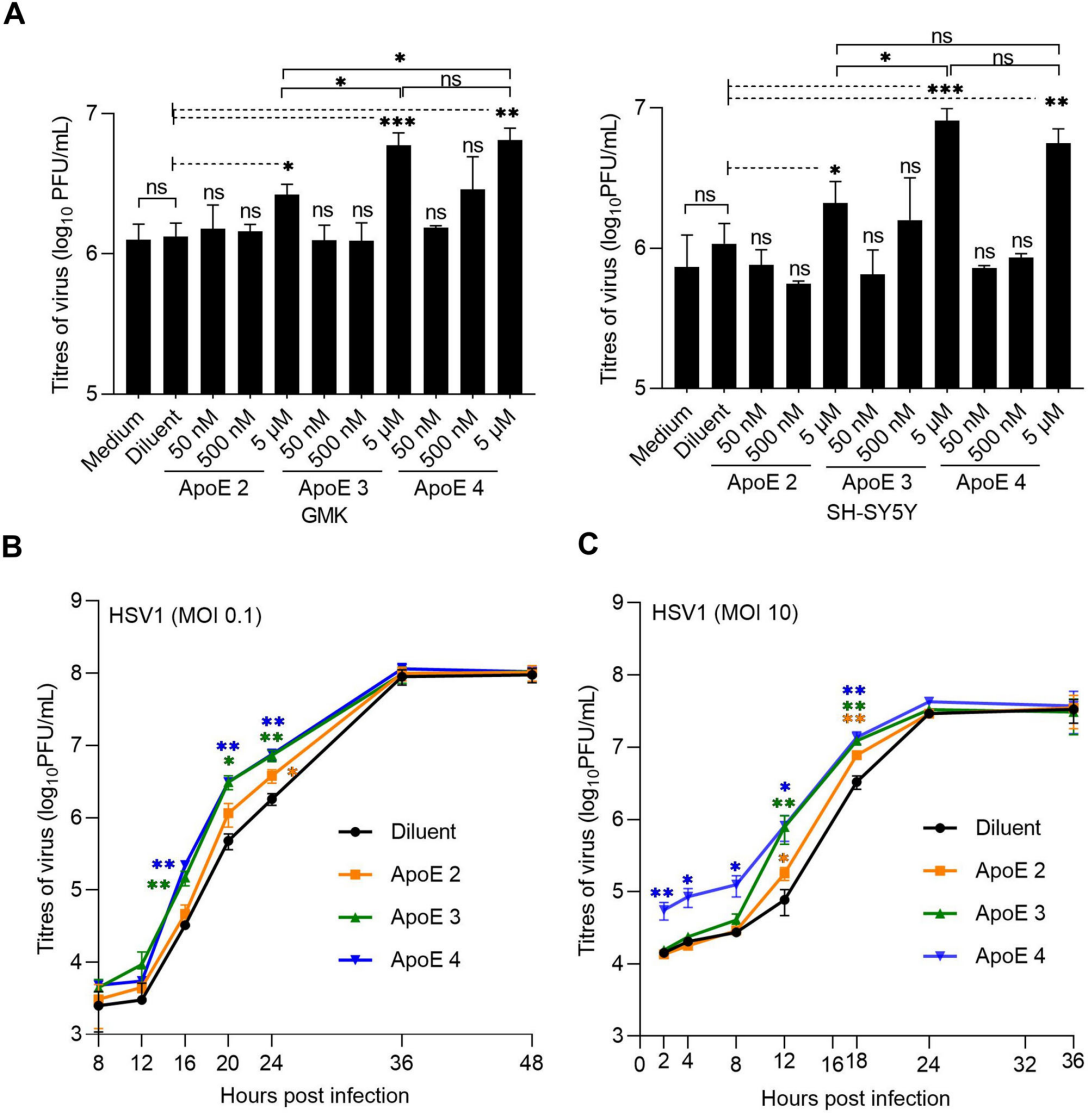
HSV1 growth is accelerated by ApoE. **A** HSV1 growth was analysed by plaque assay at various concentrations of different ApoE isoforms on GMK (left) or SH-SY5Y (right) cells. All the ApoE groups were compared to the diluent group. 5 µM of ApoE 2, 3, and 4 were compared with each other. **B**, **C** HSV1 growth kinetics of multiple (MOI 0.1) (**B**) or single (MOI 10) (**C**) infection cycles were studied on GMK with 5 µM of the different ApoE isoforms. Results represent at least three independent repeats. Error bars: mean ± SD (standard deviation). Student *t*-test, ns not significant, *p ≤ 0.05, **p ≤ 0.01, and ***p ≤ 0.001.

To study how the presence of ApoE affects HSV1 infection and explore potential differences between the allelic isoforms of the protein, we tested HSV1 growth in the presence of various concentrations of ApoE in both the neuronal blastoma cell line SH-SY5Y, and an epithelial cell line commonly used for HSV1 studies, green monkey kidney cells (GMK). In both SH-SY5Y and GMK cells, ApoE expression levels were negligible as verified by western blot (Supplementary Fig. 1). This is in line with the notion that ApoE is mainly secreted by the liver or by astrocytes in the brain^57^. HSV1 growth was thus investigated by adding ectopically expressed and purified ApoE solutions to cells. In such experiments, solutions of isoform 2, 3 or 4 with designated concentrations were prepared in cell culture mediums and then added to cells after 1 h inoculation of HSV1 at multiplicity of infection (MOI) 0.1. At 24 h post infection (hpi), viruses released in the medium were harvested and titrated by plaque assays. The results show that HSV1 released in the medium was increased with 5 µM ApoE 2, 3 or 4 both on SH-SY5Y and GMK, but not at lower concentrations of any of the ApoE isoforms (Fig. 1A). In comparison to the diluent group on GMK, 2, 4.5, or 4.8 times more HSV1 were detected in the supernatants when ApoE 2, 3, or 4 was present. The increase of the released HSV1 by ApoE was similar on SH-SY5Y, with 1.9, 7.6, or 5.2 times more virus in the presence of ApoE 2, 3, or 4. The promotion of released HSV1 by ApoE is also isoform dependent, with ApoE 3 or 4 demonstrating a higher effect than ApoE 2. There was no effect of the diluent on HSV1 growth as compared to that of the medium group, confirming that the increased virus amounts in the supernatant resulted from the presence of ApoE. Concomitantly, ApoE uptake at different concentrations was evaluated by immunofluorescence staining (Supplementary Fig. 2A), revealing that at least 2.5 µM is needed to guarantee an effective uptake of the protein by most cells, which is highly correlated to the increased quantities of HSV1 in the supernatants at these two concentrations (Supplementary Fig. 2B). HSV1 growth was also tested on SH-SY5Y cells where ApoE was present prior to and kept during the period of virus infection except for the 1 h inoculation. Promotion of HSV1 growth was observed with 5 µM ApoE 2, 3, or 4 to a similar extent as that in Fig. 1A, but not under other conditions (Supplementary Fig. 3A), suggesting that ApoE addition prior to infection was not additionally beneficial or detrimental under the investigated experimental conditions. Cell viability was tested in the presence of 5 µM ApoE 2, 3, or 4 confirming that none of the ApoE isoforms affected cell growth (Supplementary Fig. 3B,C). Considering that the phenotype is the same on both SH-SY5Y and GMK, and that GMK is commonly used for HSV1 research in the lab, which gives practical benefits to our study, we decided to work on GMK for the rest of our experiments.

Next, we investigated the HSV1 growth kinetics of multiple (MOI 0.1) or single infection (MOI 10) cycles on GMK in the presence of 5 µM ApoE isoforms. During multiple infection cycles (Fig. 1B), HSV1 in all groups had similar titers at shorter time points (<12 hpi) and reached the same plateau values. However, in comparison to the diluent group, higher virus titers were detected in the medium at middle time points (16, 20, and 24 hpi), when ApoE was present (Fig. 1B). These promotional effects were isoform dependent: At 24 hpi with MOI 0.1 or 18 hpi with MOI 10, two times more HSV1 were released in the supernatants with ApoE 2, and about four times with ApoE 3 or ApoE 4 provided (Fig. 1B). Similar as in multiple infection cycles, isoform-dependent higher virus titers were also seen at middle time points (12 and 18 hpi) for ApoE groups as compared to the diluent group during virus single infection cycle, with more viruses detected in the ApoE 3 or 4 group than in the ApoE 2 one (Fig. 1C). An interesting observation was that for the ApoE 4 case, under single infection cycle conditions (high MOI), more HSV1 viruses were detected at early time points, which most likely resulted from the release of extracellular viruses from the cell surface, instead of promoted virus growth, in view of the short time after infection initiation. Considering the similar infection starts and plateaus, these results suggest that one or multiple steps of the HSV1 lifecycle are promoted by ApoE, resulting in more released (cell-free) viral particles. This made us investigate the potential roles of ApoE at different infection steps individually. In our experimental design, purified ApoE was added after 1 h inoculation. Given that most HSV1 can successfully enter cells within the 1 h inoculation^58^, virtually all cells are infected with MOI 10 infection before the addition of ApoE. Thus, no influence of ApoE on binding and entry of the internalized HSV1 is expected for the single infection cycle case (MOI 10, Fig. 1C) and for the first round of infection of the multiple cycle case (MOI 0.1). Thus, to focus on the proviral effects of ApoE, we first directed our attention to the processes after virus entry, including virus replication and egress.

### HSV1 detachment from the cell surface, but not replication, is accelerated in the presence of ApoE

**Fig. 2:**
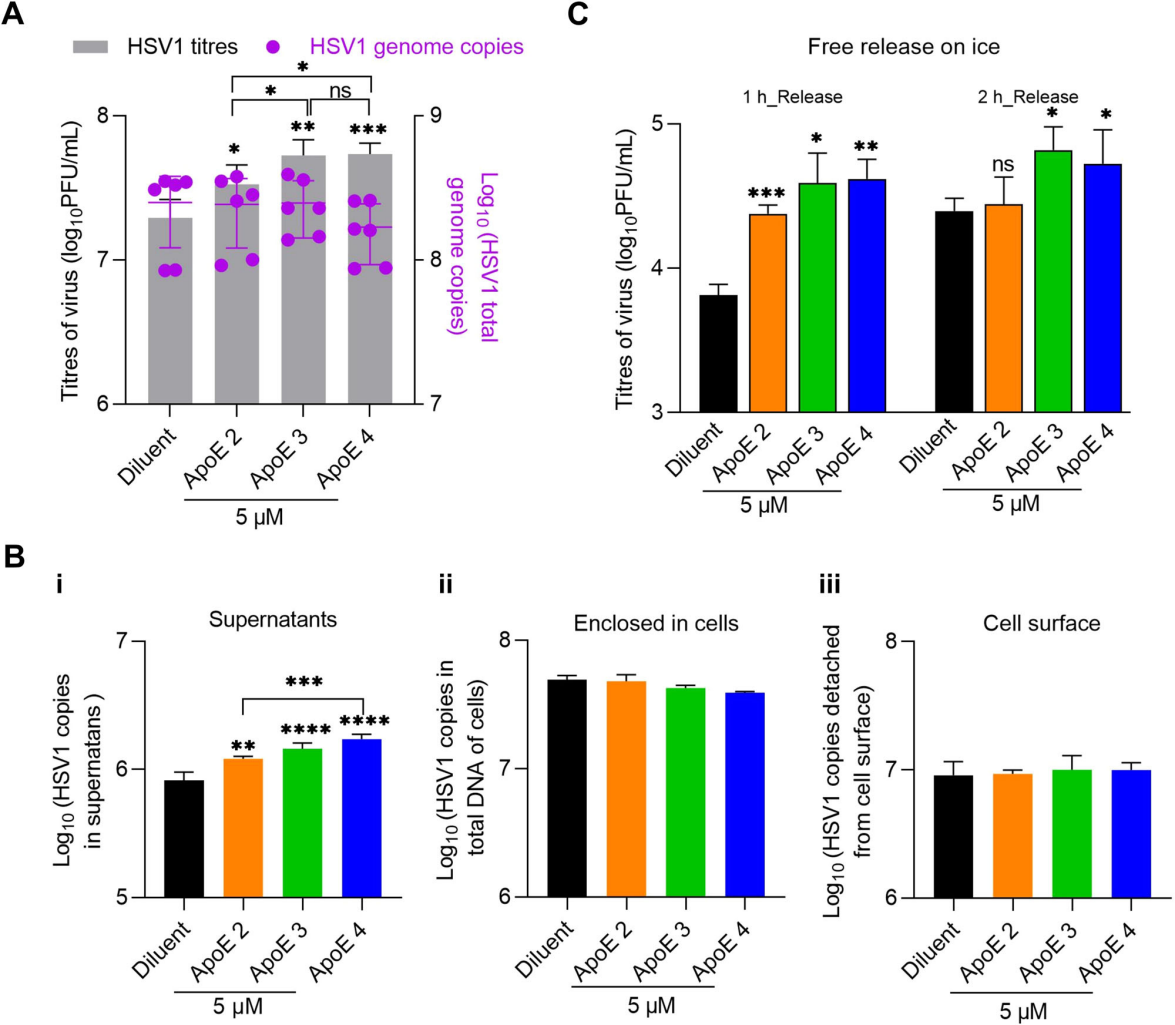
HSV1 release, but not replication, is promoted by ApoE. **A** GMK cells were infected with HSV1 (MOI 0.1) and ApoE or diluent was added at 1 hpi. At 24 hpi, medium and cell samples of different groups were separated and quantified by plaque assay for released viruses (gray bars, left Y axis) and qPCR for cell-associated ones respectively (purple dots, right Y axis). **B** Infection was done in the same way as in (**A**). At 20 hpi, HSV1 on the cell surface was released by trypsin after the separation of supernatants (medium) and cell samples as in (**A**). Supernatants (i), enclosed in cells (ii), or HSV1 released by trypsin (iii, cell surface) were quantified by qPCR. **C** Infection was done in the same way as in (**A**). At 20 hpi, the supernatant was removed, and cells were extensively washed with cold PBS. Virus release was monitored for 1 or 2 h and quantified by plaque assay. For the data analysis in (**A**, **B**), ApoE groups were compared with the diluent individually as well as with each other. The significance is indicated on the top of each column for the virus titers or genomes. No significance was observed for the statistical analysis between any group of genome copies, enclosed cells, or cell surface. Results were from three independent repeats. For the data analysis in (**C**), ApoE groups were compared to the diluent group individually at both time points. Results represent four independent repeats. Student *t*-test, ns not significant, *p ≤ 0.05, **p ≤ 0.01, ***p ≤ 0.001 Error bars: mean ± SD.

HSV1 replication was analysed by qPCR for genome quantification. At 24 hpi with HSV1 infection of MOI 0.1, supernatant and cell-associated viruses were harvested separately and quantified by plaque assays and qPCR respectively. Titration of the supernatant by plaque assays was included as a control, and the results revealed increased viral titers in ApoE-added groups (Fig. 2A, left Y axis), in agreement with previous data (Fig. 1). In contrast, cell-associated virus genome copies were similar in all groups (Fig. 2A, right Y axis). These results strongly suggest that ApoE promotes HSV1 release without affecting its replication.

Next, we quantified the amounts of viruses on the cell surface prior to release. This quantification could be used as an indication of the efficiency of virus assembly and intracellular traffic to the cell membrane. For this purpose, samples were harvested at 20 hpi. After the separation of cells and supernatants, virus particles displayed on the cell surface were released by trypsin digestion and collected. Then, the three parts of the sample, i.e., viruses in supernatants, displayed on the cell surface, or enclosed in cells were analysed. Quantification of virus in supernatants by qPCR revealed ApoE isoform-dependent virus promotions (Fig. 2B-i, supernatants), which was also observed by titration of the supernatants via plaque assay (Supplementary Fig. 4). Quantification of cell-associated viruses did not yield significant differences neither in the cell-enclosed nor trypsin-released (cell-surface) materials, suggesting that virus packaging or trafficking is unlikely modified by ApoE (Fig. 2B-ii, iii). With more viruses in the supernatant but similar amounts on the surface, our results strongly suggest that HSV1 release from the cell membrane is promoted in the presence of ApoE and that the HSV1 interaction kinetics at the cell surface reach a new equilibrium in the presence of ApoE.

The quantities of HSV1 are similar on the cell surfaces of both mock and ApoE-treated groups, as shown in Fig. 2B. Taking this as the starting point, we monitored HSV1 release from the cell surface. In this experiment, the supernatant was removed at 20 hpi, and the cells were washed with cold PBS. Subsequently, virus release was monitored by harvesting the supernatant at 1 and 2 h after washing and quantifying its viral titer by plaque assay. As HSV1 trafficking and release are highly dependent on cellular exocytosis^59^, the experiment was performed on ice, in order to inactivate exocytosis, thus excluding the transport of new particles to the cell membrane. Supernatants at 20 hpi were titrated as controls before the evaluation of HSV1 release, which confirmed that there were more viruses in the supernatants of ApoE groups at this time point. For both release time points, more viruses were detected in the supernatants from ApoE groups than that of the diluent group (Fig. 2C), leading to the conclusion that HSV1 detaches faster from the cell surface, representing cell-free release and transmission of HSV1, in the presence of ApoE. As there was no ApoE added in the medium during the release test, we speculate that when HSV1 detaches from the plasma membrane, ApoE is either enriched in the newly produced virus particles or present at the plasma membrane. These suggested scenarios are further investigated below.

### ApoE associates with HSV1 particles

**Fig. 3:**
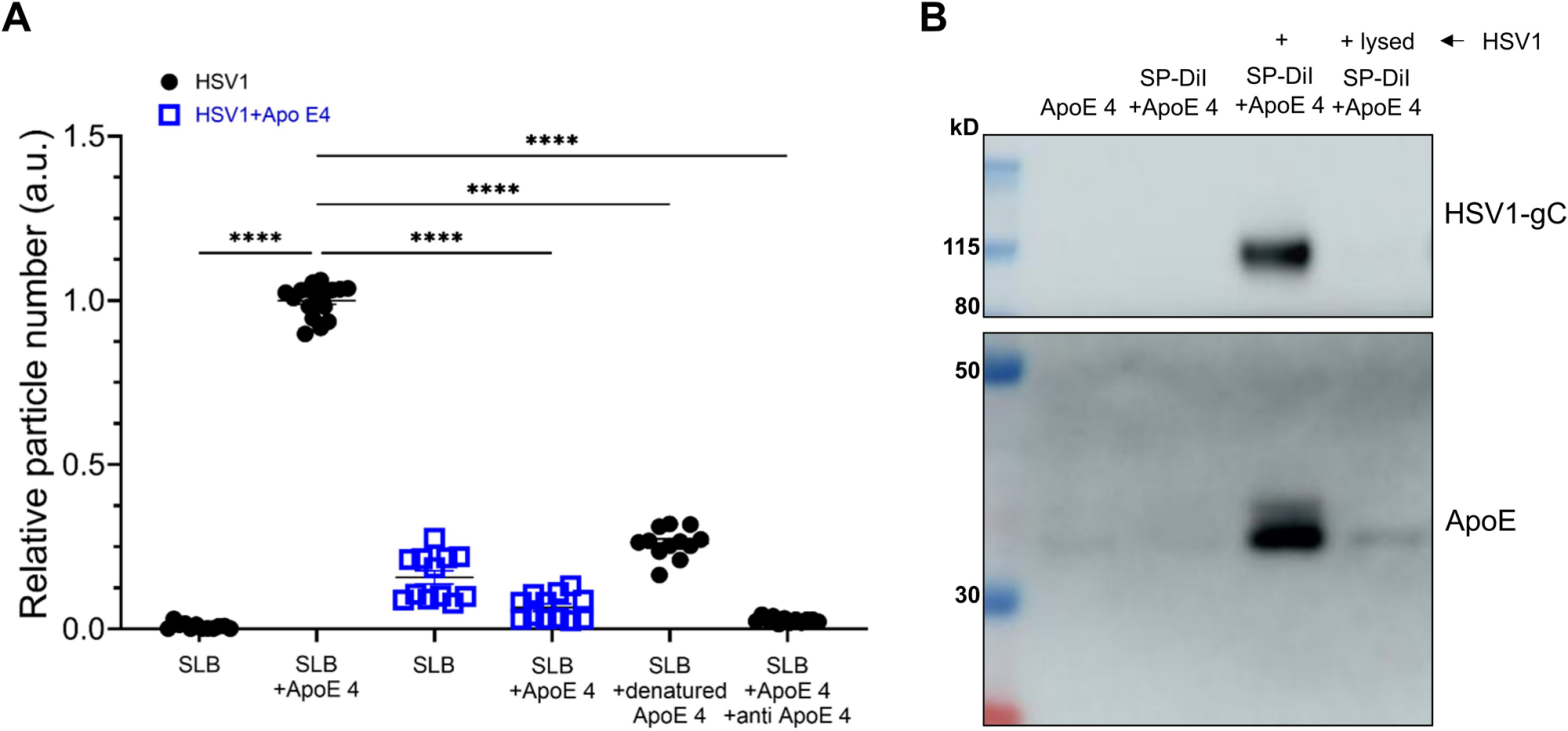
Interactions between ApoE and HSV1 particles. **A**, **B** About 2 × 10^8^ PFU of HSV1 purified via ultracentrifugation, 4 µM ApoE, and 40 µM fluorescent lipophilic dye (SP-DiI) were incubated for 1 h in PBS and separated as described in the methods section. **A** Numbers of HSV1 or ApoE 4-coated HSV1 particles on a POPC:DGS-NTA membrane surface (referred to as supported lipid bilayer (SLB) here) with or without His-ApoE 4 immobilized, or carrying denatured ApoE, as a negative control. ApoE antibodies (150 µg/mL, 1 h incubation) were also included in one case, to block HSV1-ApoE interactions. The HSV1 particles were labeled with the membrane dye SP-DiI. Each point in the plot represents particle count from a single image of 6 or 12 randomly selected fields. Data were produced from two independently prepared surfaces. Particle binding to ApoE 4 carrying bilayers (SLB+ApoE 4) was used for normalization. Statistics analysis is done by ordinary one-way ANOVA test. Error bars indicate mean and standard error of mean (SEM). ****p ≤ 0.0001. **B** Samples were lysed after incubation and separation and processed for western blot. HSV1-gC and ApoE were probed. Original blots (Supplementary Fig. 8).

HSV1 has been shown to bind various human lipoprotein particles and artificial proteoliposomes^60^. Recent studies have also revealed that ApoE enriches in virus particles produced from ApoE-expressing cells and plays important roles in efficient HBV and HCV infections^38–41^. Our results made us wonder whether ApoE could interact and thus associate with HSV1. To address this, the direct interaction between ApoE and HSV1 was analysed by binding fluorescently labeled HSV1 particles to surface-immobilized ApoE. As shown in Fig. 3A, the particles bound specifically to the surface-immobilized ApoE 4, as further demonstrated with control experiments visualizing the nearly abolished binding of fluorescent HSV1 particles to denatured ApoE 4, as well as in inhibition experiments carried out with an anti-ApoE antibody. Additionally, preincubation with soluble ApoE 4 reduces HSV1 particles binding to surface-immobilized ApoE 4 below control levels. (Fig. 3A, blue squares). It is to be noted, that particles were labeled with the membrane inserting dye SP-DiI, likely to label both HSV1 particles and a minor fraction of extracellular vesicles possibly co-purified with HSV1^61–63^ (see Supplementary Fig. 5 for further details). To distinguish HSV1 virions from extracellular vesicles and demonstrate the direct interactions between HSV1 and ApoE, HSV1 virions were detected by qPCR with a pull-down assay, in which His-ApoE 4 was immobilized on bilayer-coated beads. The qPCR quantifications further confirmed that HSV1 specifically attached to the immobilized His-ApoE 4 (Supplementary Fig. 6). To further support these results, we tested whether ApoE 4 added exogenously can bind stably to HSV1 particles and thus be co-purified together with HSV1 as a complex. In this case, a purification protocol based on size exclusion was developed to efficiently remove free protein while recovering intact particles. Western blot analysis showed that ApoE 4 was recovered after purification only in the presence of intact HSV1 particles, strongly suggesting that the protein interacts with the virus particles when added exogenously (Fig. 3B). Lysed HSV1 was included as a control, as the separation is based on the sizes of complexes; in this case, neither HSV1 nor ApoE were collected during the purification procedure. Together, these results lead to the conclusion that purified ApoE 4 specifically interacts with HSV1 particles, and that the complexes formed by ApoE and HSV1 remain stably associated after separation of free ApoE. The apparent affinity of the ApoE-HSV1 interaction was found to be isoform-dependent, with ~3 times higher apparent affinity of ApoE 4 to the virus particle as compared to ApoE 2 and ApoE 3, mostly driven by an increase in the association rate constant of the particles to the protein (Supplementary Fig. 7).

### ApoE decorated HSV1 demonstrates faster attachment and higher efficiencies of both attachment and entry to cells

**Fig. 4:**
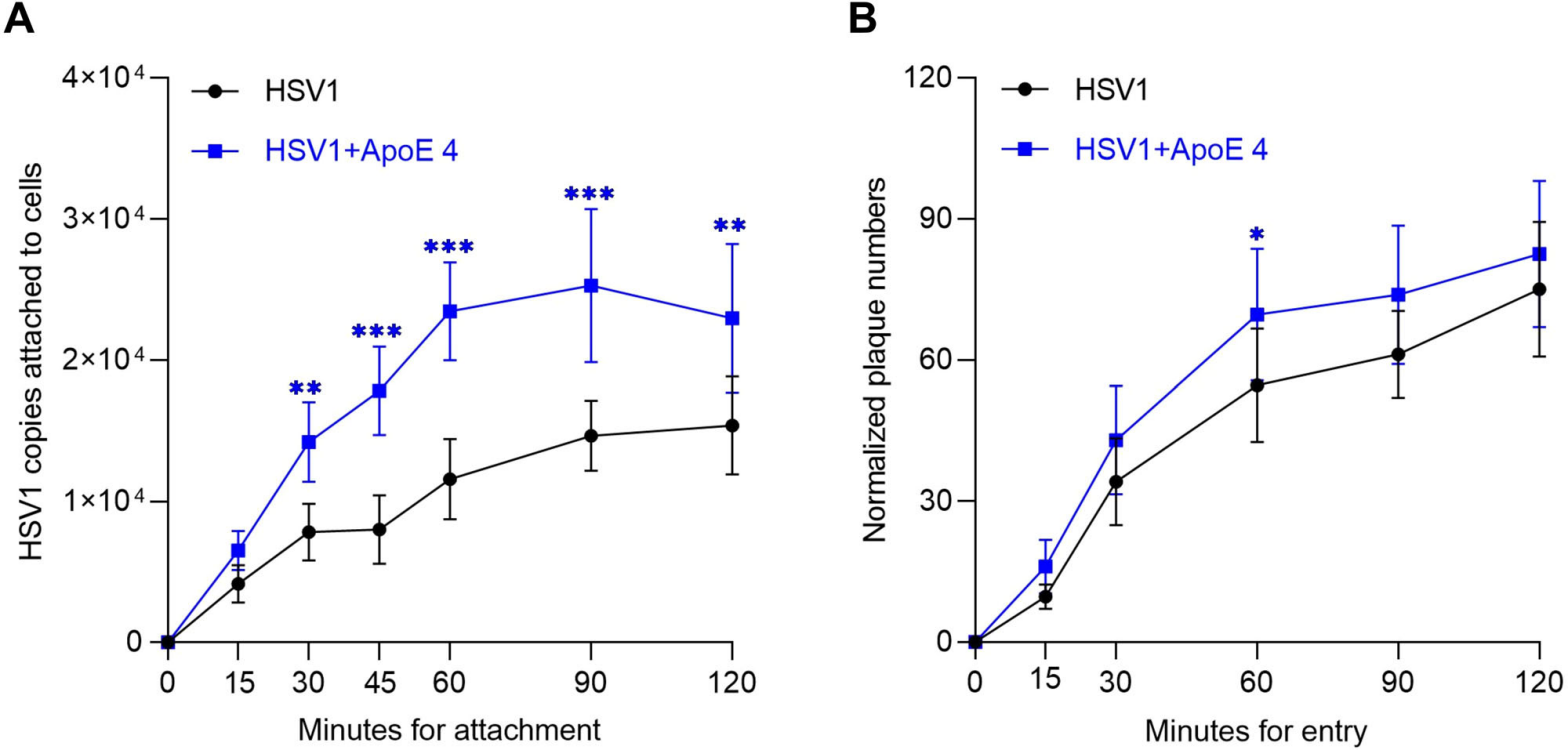
Comparison of the binding and entry of HSV1 and ApoE 4 coated HSV1. **A** The same amounts (50,000 copies of each according to the qPCR quantification) of HSV1 and ApoE 4-coated HSV1 (purified HSV1 via ultracentrifugation, prepared as in Fig. 3) were added to GMK cells for attachment by placing both the cells and the inoculum on ice. At indicated times, the attached viruses were collected and quantified by qPCR. The data represent results from three independent repeats. **B** The entry kinetics of HSV1 or ApoE 4-coated HSV1 was studied after 1 h synchronization on ice. Before entry initiation, the amounts of the attached virus after 1 h synchronization on ice were quantified by qPCR in a parallel group. The plaque numbers are presented after normalization to the amounts of attached viral copies. The results are the summary of three independent repeats. Student *t*-test, significance *p ≤ 0.05, **p ≤ 0.01, ***p ≤ 0.001 Error bars: mean ± SD.

The observation that ApoE associates with HSV1 particles raises the question of whether ApoE associated with HSV1 modifies the interactions between HSV1 and the cell surface, thereby influencing the attachment and entry process. This was tested by comparing the binding and entry of HSV1 and ApoE 4-coated HSV1 separately. To get the same input for both types of HSV1 during the binding experiment, the prepared viruses were quantified by qPCR, and the same quantities (50,000 copies) of each were added to cells for attachment on ice. Our results show that there were more copies of ApoE 4-coated HSV1 attached to the cell surface than for the ApoE-free HSV1 group at most time points (Fig. 4A), which indicates faster attachment kinetics and higher attachment efficiencies. At 60 min after attachment on ice, the quantities of ApoE 4-coated HSV1 are ~2 times higher than that of ApoE-free HSV1. To compare the entry behavior, while taking into account differences in attachment efficiency, the amounts of entered viruses (the plaque numbers) were normalized according to the amount of particles bound to the cell surface, yielding a value reflecting the propensity of an attached virus to enter into the cell (entry efficiency). These binding quantities were determined by qPCR quantification of the number of attached particles on ice, parallel to the entry experiment (see details in the methods section). As displayed in Fig. 4B after normalization, ApoE 4-coated HSV1 demonstrated slightly higher entry efficiency at all time points, (Fig. 4B), although the difference was only statistically significant 60 min after entry initiation. Taken together, our results indicate that harboring ApoE can also be beneficial to HSV1, primarily by facilitating virus attachment to the cell, which results in more entry. The direct effect on the uptake process itself and thus on entry efficiency, remained overall modest.

### The interactions between HSV1 and the cell surface are modified by virus-bound ApoE

**Fig. 5:**
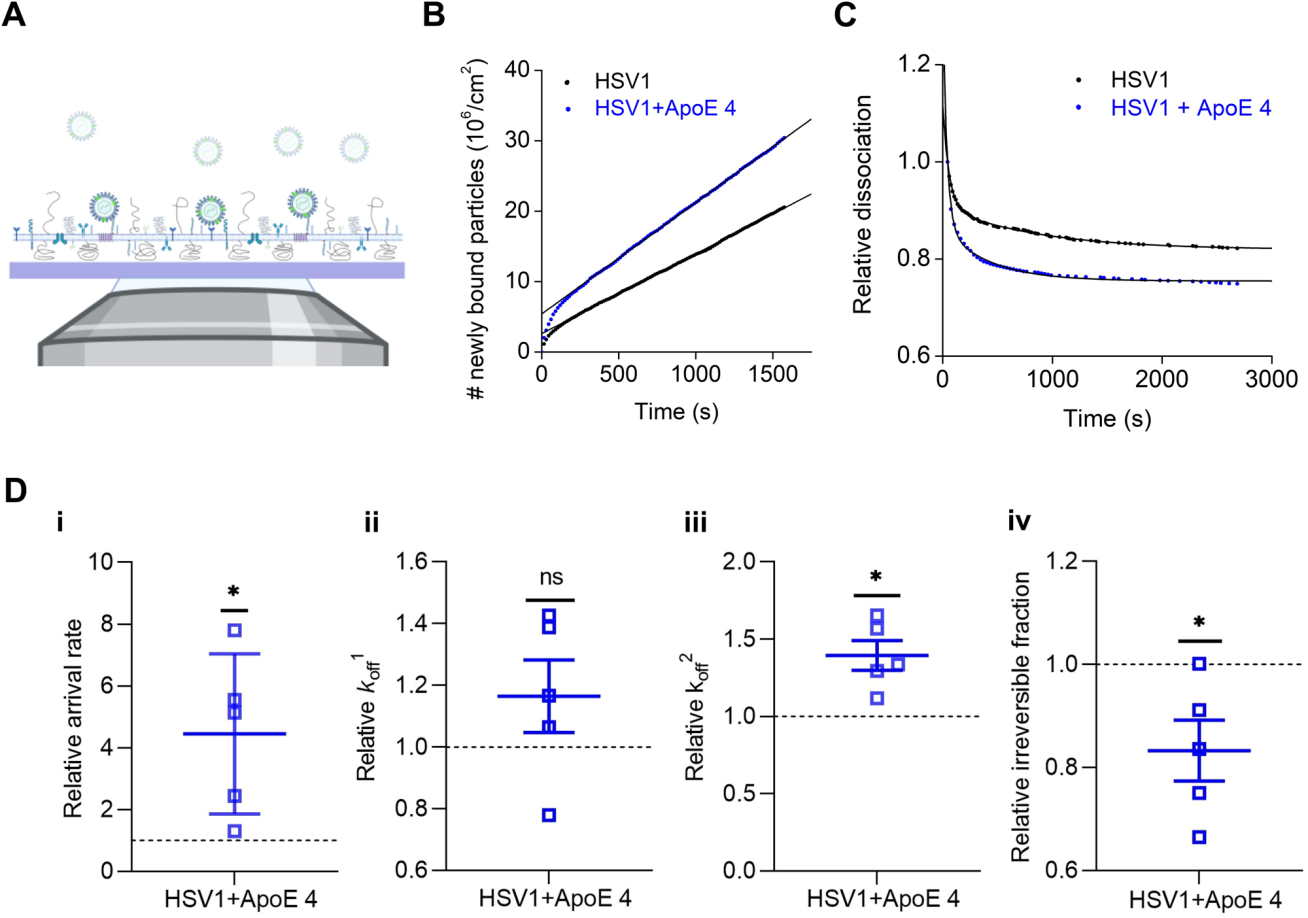
ApoE 4-coated HSV1 particles display faster attachment and detachment from the native supported bilayers. **A** Schematic representation (not to scale) of the TIRFM-based assay to probe the interaction of fluorescently labeled particles to nSLBs. Planar nSLBs are formed on a glass substrate, before being incubated with fluorescent viruses. The interaction between the fluorescent viruses and the nSLBs from GMK membranes (lacking ApoE) can then be imaged using TIRFM, selectively illuminating only viruses interacting with the surface, while ignoring free viruses in the bulk solution. Representative curves of (**B**) the association and (**C**) the dissociation kinetics of HSV1 (black) and ApoE 4-coated HSV1 (blue) to nSLBs with the corresponding linear and double exponential decay fits, respectively. **D** Mean and SEM of the relative association (**D**-i, arrival rate) from the linear fit of curves as shown in (**B**) and relative dissociation kinetics (**D**-ii, *k*_off_^1^; **D**-iii, *k*_off_^2^; and **D**-iv, irreversible fraction) from the double exponential fit with an offset, of the data as shown in (**D**) (see the methods section for details). Blue squares represent the binding data for ApoE 4-coated HSV1 particles to nSLBs, which are normalized to HSV1 data (black dotted line). Each data point is a sum of particles at three different positions per well from at least two independent experiments. Statistical significance is calculated using one sample *t*-test with mean 1 (*p ≤ 0.05). The schematic in panel (**A**) was made with BioRender.com.

The results of the facilitated attachment, entry, and release of HSV1 suggest that ApoE modifies interactions between HSV1 and the cell plasma membrane. To further characterize these interactions, we use a well-controlled biophysical experimental system, which allows us to focus on the effect of ApoE on the virus-membrane interaction, while excluding the contribution of other cellular factors. In this experiment, we characterized the interaction kinetics of HSV1 particles decorated with ApoE or ApoE-free HSV1 particles, to membranes from GMK cells, using total internal reflection flourescent microscope (TIRFM). For this purpose, native supported lipid bilayers (nSLBs)^64,65^, i.e., two-dimensional planar lipid membranes supported onto a glas substrate and containing plasma membrane material, were used as cell-surface mimetics in biophysical studies of virus-membrane interactions. TIRFM allows for the visualization of surface-bound viruses while discriminating them from the ones in solution (Fig. 5A). In this case, the particles were labeled with the lipophilic dye SP-DiI, which may result in the labeling of both HSV1 particles and co-purified extracellular vesicles. Immunostaining of fluorescent particles with a mixture of anti-HSV1 antibodies, and visualization on a single-particle level, revealed a dependency between the brightness of the SP-DiI stained particles and the presence of viral glycoproteins on their envelope. In particular, dim particles show less colocalization with the antibody signal. For this reason, we carefully selected an intensity threshold (see Methods for further details) to exclude dim particles and, under these conditions, estimated that the majority of the particles (70 ± 9%) considered in the analysis were virus-like and carried both lipid membrane and viral glycoproteins (Supplementary Fig. 5D). Independent colocalization experiments carried out by staining the major capsid protein VP5 of HSV1, further confirmed the presence of majority of virus particles (Supplementary Fig. 5C).

Particle visualization with TIRFM at equilibrium conditions enables the analysis of virus-membrane interaction kinetics on a single-particle level using an experimental procedure referred to as equilibrium fluctuation analysis (EFA)^66^. In EFA, the association of viruses to the surface can be plotted over time (Fig. 5B) and the arrival rate, directly proportional to the association rate *k*_on_, can be quantified, provided that the experiment is carried out under reaction-limited conditions. Additionally, information on the dissociation behavior of the virus from the membrane can be obtained by analysis of the particle residence time; fitting the dissociation curves with a double exponential decay function with an offset (Fig. 5C) allows for a quantification of the apparent dissociation rate constants (*k*_off_) (Fig. 5D)^65^ as well as of an irreversible fraction determined from the offset value (see the methods section for details). These experiments revealed that ApoE 4-coated HSV1 associated significantly faster to the membrane, showing an average 4.4 ± 2.6 times faster arrival rates than noncoated HSV1 (Fig. 5D-i). The virus also exhibited a heterogeneous detachment behavior; virus dissociation could broadly be separated into two dissociating populations characterized by fast (*k*_off_^1^) or slow (*k*_off_^2^) dissociation rates and a population of irreversibly bound (non-dissociating) particles. On average, 85% (ApoE 4-free) and 77% (ApoE 4-coated) of the particles were bound irreversibly (Supplementary Table 1), while the fit revealed that a roughly equal fraction of the remainder dissociating particles belong to *k*_off_^1^ resp. *k*_off_^2^, in all cases (Supplementary Table 1). The values of *k*_off_^1^ were, on average, 0.023 s^-1^ for HSV1 and 0.027 s^-1^ for ApoE 4-decorated HSV1: they were 0.0010 s^-1^ and 0.0011 s^-1^, respectively for *k*_off_^2^ (Supplementary Table 1). The values for the slow dissociating fraction are comparable with what was observed in a similar experimental setup for the interaction of HSV1 to GMK cells^65^. From these values we can estimate the unbinding energy for the two subpopulations assuming for simplicity a single energy barrier with logarithmic kinetics^66^. Under these assumptions, the binding energy (*E*_B_^*n*^) can be calculated from *k*_off_^*n*^. as:

$${{E}_{{\rm{B}}}}^{n}=-{k}_{{\rm{B}}}T\,{\mathrm{ln}}\left({{k}_{{\rm{off}}}}^{n}/A\right)$$

Where ${k}_{{\rm{B}}}$ is the Boltzmann constant, T is the absolute temperature and A is a constant indicating the attempt rate and usually in the order of 10^13^ s^-1^. The value of *E*_B_^1^ is in the order of 83 kJ/mol while *E*_B_^2^ is 90 kJ/mol. Thus, the energy reduction due to the presence of ApoE, which is independent of the value of A, is 0.37 kJ/mol for the fast component and 0.88 kJ/mol for the slow one.

Although no significant difference was observed for the fast dissociation rate constant (Fig. 5D-ii, *k*_off_^1^), ApoE 4-decorated HSV1 particles were found to exhibit 39% higher dissociation rates for the slower dissociation rate constant compared to HSV1 (Fig. 5D-iii, *k*_off_^2^). The ApoE 4-decorated particles also show an 8.3% reduction of irreversibly bound particles compared to HSV1 (Fig. 5D-iv, irreversible). Combined, these results indicate that ApoE-decoration of HSV1 enhances the dynamics of virus-membrane interaction by increasing both the association and dissociation of viral particles from the membranes.

### ApoE 4-coated HSV1 exhibits increased association and dissociation with heparan sulfate

**Fig. 6:**
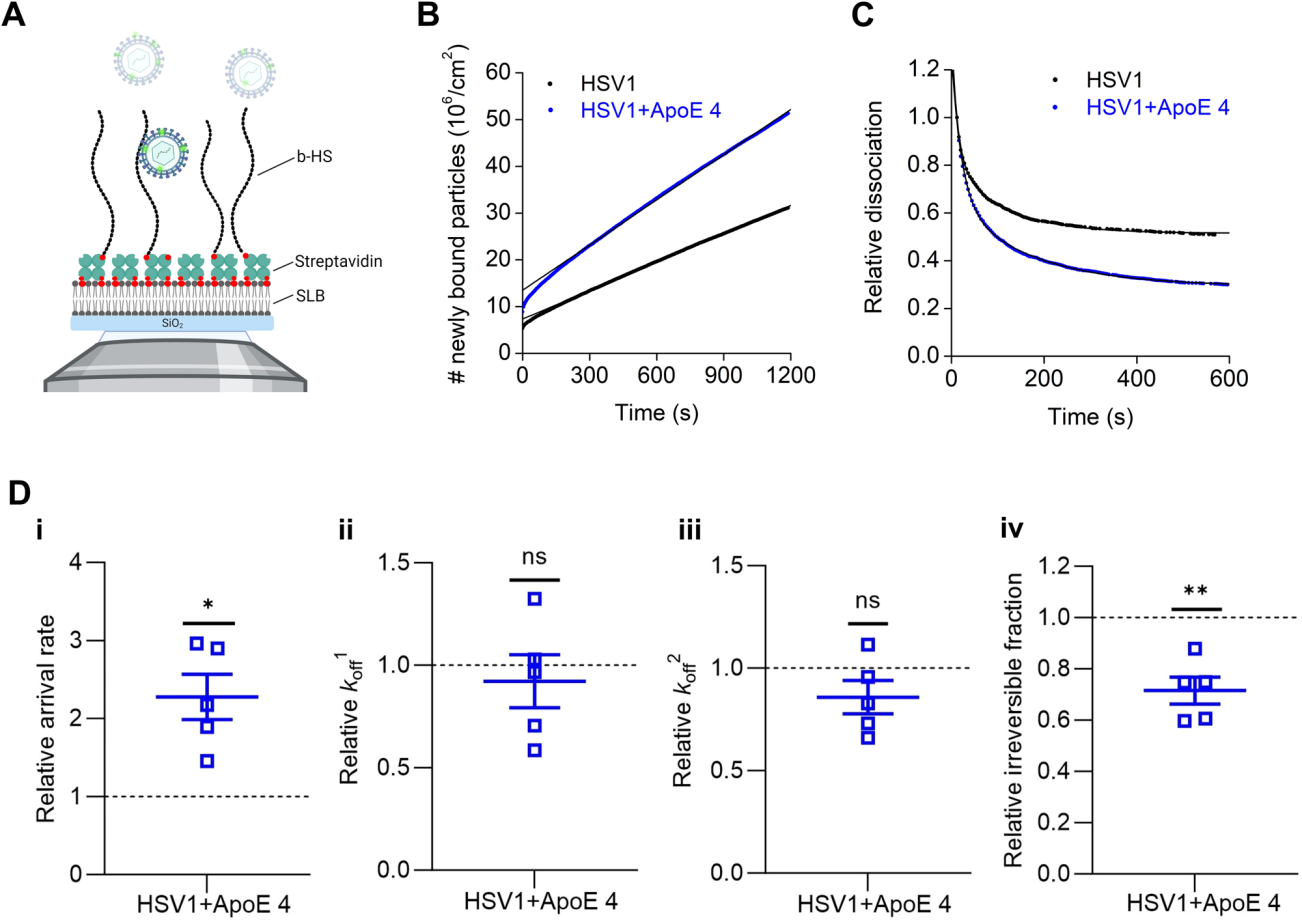
ApoE 4-coated HSV1 attaches faster to heparan sulfate. **A** Schematic representation (not to scale) of the TIRFM-based assay to probe the interaction of fluorescently labeled particles to surface-bound HS on SLBs using a streptavidin layer as a sandwich between SLBs and biotinylated HS (b-HS). Representative curves for (**B**) the association and (**C**) the dissociation kinetics of fluorescently labeled HSV1 (black) and ApoE 4-coated HSV1 (blue) particles to HS films with the corresponding linear and double exponential fits, respectively. **D** Mean and SEM of the relative association kinetics (**D**-i, arrival rate) from the linear fit of the curves as shown in (**B**) and relative dissociation kinetics (**D**-ii, *k*_off_^1^; **D**-iii, *k*_off_^2^; and **D**-iv, irreversible fraction) from the double exponential fit with an offset of the data as shown in (**C**) (see the methods section for details). Blue squares represent the binding data for ApoE 4-coated HSV1 particles to HS, which are normalized to HSV1 data (black dotted line). Each data point is a sum of particles at three different positions per well from three independent experiments. Statistical significance is calculated using one sample *t*-test with mean 1 (*p ≤ 0.05 and **p ≤ 0.01). The schematic in panel (**A**) was made with BioRender.com.

In view of the reported interaction between ApoE and HS^55^, we hypothesized that the modified interactions between HSV1 and the cell plasma membrane resulted from the interactions between HSV1 and HS on the cell surface. HS is abundantly expressed on the surface of GMK cells^67^ and also found in nSLBs derived from such cells^65^. The hypothesis was tested by looking at the binding of HSV1 and ApoE 4-coated HSV1 particles to HS. Using TIRFM measurements on surface-bound HS (Fig. 6A–C), the ApoE 4-coated particles showed on average 2 times higher association rate to HS as compared to non-coated particles (Fig. 6D-i). Although both ApoE 4-coated HSV1 and HSV1 particles detached with similar fast (*k*_off_^1^) and slow (*k*_off_^2^) dissociation rates from HS (Fig. 6D-ii–iii), a significant reduction (28%) of irreversibly bound particles was found for ApoE 4-coated HSV1 as compared to ApoE-free HSV1 (Fig. 6D-iv). To further strengthen our hypothesis that ApoE primarily influences the interaction with HS at the cell surface, we also carried out binding experiments on cells after treating them with heparinase. In such experiments, HSV1 binding was greatly decreased (>90%, as expected^32^), and the binding advantage of ApoE-coated HSV1 was abolished (Supplementary Fig. 9).

Altogether, these results indicate that ApoE 4 promotes both association and dissociation of HSV1 on HS films. In conjunction with the nSLBs results (Fig. 5), this implies that the presence of HS in the nSLBs might be a dominant factor regulating the interaction between HSV1 and HS through ApoE.

### HSV1 particles have similar dissociation rate constants (*k*_off_) on membranes with or without ApoE association

**Fig. 7:**
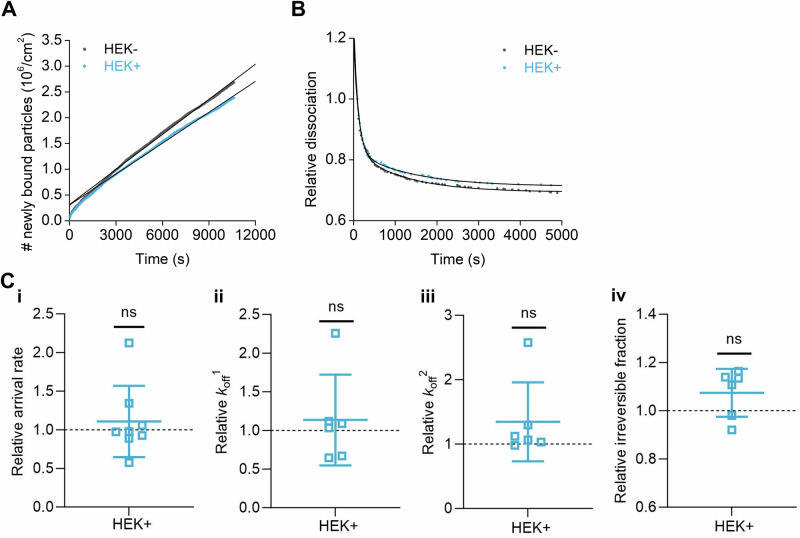
HSV1 detaches with similar *k*_off_ on native-supported lipid bilayers in the presence or absence of ApoE. Representative curves of (**A**) association and (**B**) dissociation kinetics of fluorescently labeled HSV1 from nSLBs generated from the membrane materials of HEK cells without (HEK-, black) or with (HEK+, cyan) the induction of ApoE 4, with their corresponding linear and double exponential fits, respectively. **C** Mean and SEM of the association kinetics (**C**-i, relative arrival rate) from the linear fit of the data as shown in (**A**) and dissociation kinetics (**C**-ii, iii, and iv, relative *k*_off_^1^, *k*_off_^2^, and irreversible fractions, respectively) from the double exponential fits with an offset of the data as shown in (**B**) (see the methods section for details). Cyan squares represent the binding data of HSV1 particles to nSLBs with the induction of ApoE 4 (HEK+), which are normalized to the binding data of HSV1 particles to nSLBs without the induction of ApoE 4 (HEK-, black dotted line). Each data point is a sum of particles at three different positions per well from three independent experiments. Statistical significance is calculated using one sample *t*-test with a mean of 1 (ns not significant).

We also hypothesized that HSV1 would detach faster from membranes harboring ApoE. The residence of ApoE in the plasma membrane, together with caveolin, has been reported in adipocytes^68^. Thus, we first tested whether ApoE was also membrane-associated in cells of relevance to our experiment. For this purpose, an ApoE 4 expression inducible cell line was established. The induction of ApoE 4 expression was verified by western blot (Supplementary Fig. 10A); and the presence of ApoE 4 was also confirmed in the plasma membrane materials isolated after sucrose purification (Supplementary Fig. 10B). To compare the influence of membrane-bound ApoE on virus binding kinetics, we produced nSLBs, from both ApoE 4-induced and non-induced cells. Kinetic analysis (Fig. 7A, B) with TIRFM revealed that the presence of ApoE in the membrane does neither influence the association (Fig. 7C-i) nor the dissociation behavior (Fig. 7C-ii–iv) of the virus from the membrane with or without the presence of ApoE. These results indicate that membrane-associated ApoE is not a significant factor in promoting HSV1 attachment or release.

## Discussion

In this work, we investigated the potential influence of the human protein ApoE on primary virus infection, at a molecular level. We monitored HSV1 growth on cultured cells in the presence of different ApoE isoforms and analysed the effects of ApoE on different stages of the HSV1 infection cycle. HSV1 infection was evaluated after adding exogenously expressed and purified ApoE to GMK and SH-SY5Y cells with negligible ApoE expression (Supplementary Fig. 1A). Overall, we found that HSV1 propagation is advanced when the virus is grown in the presence of ApoE. Our detailed investigations further demonstrate that the presence of ApoE facilitates the release of HSV1 from the cell surface. Furthermore, association of ApoE to HSV1 facilitates the attachment of the virus to the plasma membrane. Both processes explain the observed accelerating effects of HSV1 infection and point toward the involvement of the human protein in the extracellular spread of the virus.

The promotion of HSV1 by ApoE is concentration-dependent and was primarily observed in our system at concentrations in the micromolar range (Fig. 1A, B). Such a dependence is likely related to the uptake efficiency of ApoE by cells in our artificial in vitro system, as it was shown that ApoE addition at 2.5 or 5 µM is needed to guarantee an effective uptake of the protein by most cells (Supplementary Fig. 2), which is highly correlated to the promoted HSV1 growth at these two concentrations. Importantly, cell metabolism was not altered when this concentration of ApoE was added (Supplementary Fig. 3B, C), which implies that the promotion of HSV1 is not an artifact or an indirect effect resulting from a change in cell behavior. Such concentrations are in the same order as ApoE concentrations in the medium of Huh-7 cells (expressing ApoE 3) in culture (Supplementary Fig. 11), the cell line commonly used for studies on hepatitis viruses and ApoE, supporting our choice. Studies have reported varied physiological concentrations of ApoE, depending on the body fluid type as well as on the applied measurement technique. Reported values typically range from 0.1 to 1.75 µM^69–75^, although it cannot be excluded that ApoE concentrations are higher locally. In particular, it has been observed that local ApoE levels can change in response to stimuli, e.g., in the case of injury^76,77^, where an accumulation of ApoE in the regeneration sites can last over a month, and the peak of ApoE increase can reach over 250-fold^77^.

Incorporation of ApoE into virus particles has been reported for HBV and HCV^41,42^, with consequences on the virus attachment and detachment behavior. Regarding the attachment of those viruses, ApoE has been suggested to work as a bridge ligand between virus and cell-surface receptors, including HS^43,44,78^, the viruses themselves lacking HS-binding capacity. In addition to modifying virus interactions at the cell surface by affecting attachment to HS, ApoE has also been proposed to be involved in the late steps of HCV infection^40,45–47^, although whether HCV release is affected by ApoE is unknown. Our study reports that ApoE also associates with HSV1 through direct biomolecular interaction between ApoE and the surface of the HSV1 particle. (Fig. 3 and Supplementary Fig. 6). These results are complementary to a previous study reporting interactions between HSV1 and purified serum lipoparticles or artificial proteoliposomes harboring apolipoproteins, including ApoE^60^. ApoE distributes across the cytoplasm and the cell plasma membrane (Supplementary Fig. 13A); it can, therefore, be inferred that HSV1 may accumulate ApoE after its replication and packing in the nucleus, i.e., during viral particle envelopment, cytoplasmic trafficking, and budding on cell plasma. An association can also occur after virus release and such an association with exogenous ApoE appears to be specific, as it can be prevented by the presence of anti-ApoE antibody (Fig. 3A) and stable over several hours as concluded from the lack of binding of ApoE-coated particles to surface-immobilized ApoE (Fig. 3A), even several hours after purification of the complexes. Interestingly, this association was found to be isoform-dependent, with ~3-fold increase of attachment rate for ApoE 4 to the virus particle as compared to ApoE 2 and ApoE 3 (Supplementary Fig. 7). An intriguing, yet still unanswered question, concerns the viral biomolecules mediating the interactions between ApoE and HSV1 particles. Such interactions are likely mediated by viral component(s) on the surface of the virus particle, either viral glycoproteins or the lipid membrane of the virus. From our attempts at revealing the molecular binding partner on HSV1, we have excluded potential interactions between ApoE and some HSV1 glycoproteins, including glycoprotein B, C, D, and E, by immunoprecipitation test (Supplementary Fig. 12). These results indicate that the protein is likely to interact directly with the lipid envelope, in agreement with reports indicating that the lipid-free form of ApoE interacts with model lipid membranes of simple composition^79^. However, it cannot be excluded that other viral glycoproteins not investigated here can also act as ApoE interaction partners.

A significant promotion of released virus particles in the presence of ApoE was observed both in single (Fig. 1C) and multiple-cycle (Fig. 1B) experiments. Noteworthy, is that the promotional benefits are isoform-dependent, with higher efficiency for both ApoE 3 and 4, and in contrast to what was observed for HCV^45^. Initial infection experiments were carried out by adding ApoE after 1 h virus inoculation, followed by rinsing, an experimental setup which does not consider a putative effect of ApoE on virus attachment and entry during the first round of infection, given that HSV1 entry into GMK cells is expected to occur within minutes^58^. This indicates that the proviral effect is likely ascribed to later steps in the virus replication cycle. Careful investigation of the different steps leading to the production of progeny viruses reveals that ApoE significantly promotes virus detachment from the cell surface, as shown by experiments on live cells following the release of HSV1 into the supernatant in the absence of exocytosis (Fig. 2C). An increased detachment is also in line with biophysical experiments detailing the interaction kinetics of individual virus particles with isolated plasma membranes (Fig. 5).

The release process of a virus from the plasma membrane is generally much less studied in comparison to other steps. Several studies on this topic have pointed to the effects of viral proteins^28,33,80^ and/or host factors^35,81^ on virus release. Most of these studies stress that efficient HSV release relies on fine-tuning and overcoming the interactions between viral proteins and GAGs on the cell surface. We speculate that this may also be the case here, considering that HS is the primary interaction partner of HSV1 to the plasma membrane of GMK cells^32^ (Supplementary Fig. 9) and in line with our observation that ApoE-carrying HSV1 also dissociates faster from isolated surface-immobilized HS molecules (Fig. 6). While the molecular mechanism behind this increased dissociation remains to be elucidated in detail, we speculate that addition of ApoE to the virus particle influences the multivalent interaction by interfering, possibly through steric hindrance, with the creation of high-affinity bonds with the viral glycoproteins^82,83^.

Alpha herpesvirus particles are transported in membrane vesicles (reviewed in ref. ^84^) to specific sites on the cell surface^85,86^ via cellular exocytosis pathways. The actual delivery is completed via membrane fusion between the virus-containing vesicles and the cell plasma membrane. It is unknown whether the final release happens at the sites where virus particles become exocellular or at different sites which requires lateral movement/transport of extracellularly exposed virus particles from the membrane fusion sites. In either case, the potential interventions from ApoE or other host factors should occur at the actual virus release sites. With this assumption, it can be speculated that hijacking ApoE by the virus would be a smarter solution to localize proper amounts of ApoE at the release site than having ApoE homogeneously distributed in the plasma membrane. This may explain why we have observed improved virus detachment with ApoE-coated HSV1 (Fig. 5), but not for noncoated HSV1 on nSLBs harboring ApoE (Fig. 7). It is worth mentioning that the nSBLs were generated from non-infected cell materials (HEK or HEK-ApoE). Thus, our tests cannot exclude the possibility that virus infection modifies and re-arranges membrane association of ApoE in a way that facilitates final release.

The realization that ApoE binds stably with HSV1, even when added exogenously, prompted us to further investigate whether this association can contribute, positively or negatively, to the observed proviral effect. Such an influence becomes more important in the multiple infection cycle experiment (Fig. 1B), where ApoE-coated progeny virions are involved in subsequent cellular infection cycles. Virus attachment and entry experiments reveal that the presence of ApoE on HSV1 promotes virus attachment to, and entry into cells (Fig. 4). Promoted attachment is further confirmed by biophysical experiments revealing an increased association rate constant (*k*_on_) for ApoE-coated particles as compared to the naked ones. This effect is also likely to be related again to the initial interaction of the virus with HS on the cell surface, which acts as the main recruitment factor on the GMK cells used here (Supplementary Fig. 9) and which is a known receptor for both ApoE^55^ and HSV1^26^. Indeed, harboring ApoE can provide extra ligands to HS for HSV1 attachment to cells on the premise that the viral ligands to HS are not overlapping with the ones to ApoE. This appears to be the case for HSV1 since both gB and gC of HSV1 have been proposed to bind HS^82,83^ but have not been found to mediate HSV1 association of ApoE (Supplementary Fig. 12). As a result, we propose that ApoE-coated HSV1 has a higher binding avidity to HS due to an increased number of binding sites and that this gives the virus advantages of the faster and more efficient attachment to cells (Fig. 4). This idea is strongly supported by the kinetic studies of HSV1 binding to purified HS, where we observed faster association of ApoE 4-coated HSV1 to the immobilized HS (Fig. 6D). Taken together, with the faster detachment discussed above, we speculate that the presence of ApoE significantly impacts the molecular characteristics of the interaction between HSV1 and GAGs by increasing the number of binding sites, while reducing the average affinity of the single bonds formed.

When considering the subsequent infection rounds in the multiple infection cycle experiment, it is worth to mention that the presence of HS-bound ApoE on the cell surface, maybe under specific conditions, including high ApoE concentrations^52^ and the addition of ApoE prior to HSV1 infection (Supplementary Fig. 13B), also lead to virus binding inhibition through competition^52^, and prevent cell-free virus transmission. Virus entry efficiency was not affected when cells were exposed to ApoE prior to infections (Supplementary Fig. 13C, D). Most importantly, such an inhibition process did not dominate in our multiple cycle infection experiment, given the pronounced acceleration of HSV1 infection reported in Fig. 1. It is also worth to consider that HSV1 can employ both cell-free and cell-to-cell transmission^87–89^. In the latter case, HSV1 transmission bypasses the initial attachment to the cell surface, the step hindered by HS-bound ApoE.

In our study, we observed an isoform-dependent proviral effect, with ApoE 3 and 4 demonstrating more efficient proviral effects than ApoE 2 (Fig. 1). These isoform-dependent effects correlate with the binding affinities of the different ApoE isoforms to heparin, which inspired us to assume that virus-bound ApoE manipulates HSV1-HS interactions. Indeed, the binding affinities of ApoE to heparin vary among different isoforms: ApoE 4 and 3 have similar affinities which are higher than ApoE 2^90^. In addition to this, we report that ApoE 4 associates more firmly than ApoE 2 and 3 to the HSV1 virion (Supplementary Fig. 7), an observation which may further contribute to explain the increased proviral effect of ApoE 4. It is important to note that only ApoE 4 is a recognized risk factor for Alzheimer’s disease, whereas ApoE 3 is not. Thus, the observation that ApoE 3 and ApoE 4 contribute similarly to the proviral effect, does not align with the hypothesis that ApoE 4 alone facilitates the development of Alzheimer’s disease by increasing HSV1 infection.

The findings that ApoE 4 facilitates attachment or release of HSV1 from the cell surface indicate that ApoE primarily facilitates infection in the context of cell-free spreading. ApoE is naturally expressed and secreted by epidermal keratinocytes^91^, which indicates that the promotional effects of ApoE presented here may also be of relevance during primary infection in the epithelium. Nevertheless, HSV1 shedding from oral epitheliums is not affected by ApoE^92^. We speculate that the proviral effect associated with increased attachment to the cell surface may be relevant to virus transmission from epidermal keratinocytes where ApoE is naturally expressed and secreted^91^ to sensory neurons and retrograde transportation along the axons to the cell body of neurons^93^ where ApoE is generally lacking. In addition, ApoE-mediated increased infection in the context of an acute lytic infection in the brain, characterized by the presence of a high load of extracellular viruses, may also be of relevance in the case of HSV1-associated meningitis or encephalitis in the brain^94^.

HSV1 virus preparations purified from the supernatant of infected cells, are well-known to contain extracellular vesicles, even after careful purification^31,61^, with potential biological consequences. For this reason, we have taken great care in characterizing the presence of extracellular vesicles and assessing their influence on the obtained results and formulated hypotheses. Characterizations of our purified virus preparations reveal that our protocol collects the most concentrated HSV1 fraction (Supplementary Fig. 14A, B) and excludes large fractions of extracellular vesicles, as shown by looking at three common extracellular markers in Western blot experiments (Supplementary Fig. 14C). Extracellular vesicles are indistinguishable from viral particle in our microscopy experiments, due to the labeling with a non-specific lipophilic dye SP-DiI. To address this issue, we performed a closer evaluation of the extracellular-vesicle content through single-particle-level colocalization experiments, in which labeled viruses were further immunostained for viral glycoproteins (Supplementary Fig. 5A, B) or for the capsid protein VP5 (Supplementary Fig. 5C). Our characterizations reveal indeed the presence of a minor fraction of extracellular vesicles whose impact in our single-particle kinetic assays can be minimized through careful selection of the analysis parameters. While the presence of extracellular vesicles remains a limitation of the study and while it cannot be excluded that this fraction affects to some extent the observed interaction kinetics, the good qualitative agreement with biological experiments suggests that such effects are relatively marginal. Furthermore, it is important to note that the presence of some extracellular vesicles would not invalidate any of the biological conclusions obtained on cell assays. Indeed, these biological assays employing plaque assay or qPCR quantification rely on infectious particles or require the detection of the viral genome; they are thus insensitive to the presence of most extracellular vesicles. We therefore conclude that the ApoE functions revealed in this work are unlikely influenced by extracellular vesicles. Since anti-viral^61,63^ or proviral^95–97^ effects of extracellular vesicles on HSV1 infection have been reported in the literature, investigation of the interplay between ApoE, HSV1 infection, and extracellular vesicles represents a compelling research question beyond the scope of this work; it requires further investigations with suitable experimental procedures.

In conclusion, our results reveal novel roles of ApoE during HSV1 infection at molecular levels, together with isoform-dependent effects. These ApoE-associated proviral effects may have important implications in the development of various HSV1-associated pathologies. However, the consequences of the reported molecular-level connection between ApoE and HSV1 infection on the development of Alzheimer’s disease remain elusive. Here, we have limited ourselves to a simplified model using selected immortalized cell lines and purified ApoE and HS. While our results clearly conclude a proviral effect of ApoE on HSV1 infection and can deduce interesting insights on the importance of ApoE and how it shapes infection, our model system lacks the complexity of a true biological system that could possibly mask or alter the resulting effects. As a follow-up, more clinically relevant and comprehensive models, such as epidermal keratinocytes and recently developed brain organoids^98,99^, are suggested. These will allow further and deeper studies of ApoE and HSV1 interactions, and make it possible to better assess their significance in disease development. Moreover, the interaction with other factors, such as amyloid-beta and tau protein should be considered, as the development of Alzheimer’s disease is a complicated process.

## Methods

### Cells, ApoE proteins, glycosaminoglycan, and other proteins

Green monkey kidney (GMK AH-1) cells were kindly provided by Tomas Bergström (Gothenburg University), which has been originally described previously^100^. Neuroblastoma SH-SY5Y (CRL-2266) cells were purchased from ATCC, and kindly provided by Niklas Arnberg (Umeå University). The hepatocyte-derived carcinoma cell line (Huh-7) was a gift from Magnus Evander (Umeå University). GMK and Huh-7 cells were cultured in DMEM (D5648-10L, Sigma), supplemented with 10% fetal bovine serum (FBS, SV30160.03, cytiva), 20 mM HEPES, penicillin (0.5 unit/mL) and streptomycin (50 µg/mL) (P0718, Gibco). SH-SY5Y cells were cultured in a mixture of medium with 1:1 DMEM + F-12 HAM (21700-075, Gibco), 10% FBS, 20 mM HEPES, penicillin (0.5 unit/mL), and streptomycin (50 µg/mL). The ApoE 4 inducible cell line was established by using the Flp-In ^TM^ T-Rex ^TM^ system (a kind gift from the Anna Överby group, Umeå University), according to the manufacturer’s instructions (Invitrogen). In brief, the ApoE 4 gene was synthesized and verified by the Protein Expression Platform at Umeå University, and then constructed in the backbone of pcDNA™5/FRT/TO expression vector. Together with a recombinase expressing vector pOG44, pcDNA™5/FRT/TO-ApoE 4 was co-transfected into a commercially established Flp-In™-293 cell line (based on HEK-293) carrying the integrated FRT. After transfection, selection for successful gene integration was done under hygromycin (100 µg/mL). The ApoE 4 inducible cell line was derived from a single clone and the induction of ApoE 4 expression by tetracycline (1 µg/mL) was verified by western blot. Cell cultures were maintained at 37 °C and 5% CO_2_ in an incubator. Lyophilized recombinant ApoE 2 (AE-100-10), 3 (AE-101-10), and 4 (AE-102-10) were obtained from AlexoTech AB (Umeå, Sweden). To prepare the protein solution, lyophilized ApoE proteins was first dissolved in 20 mM NaOH and then mixed with 10× PBS (phosphate buffer saline; Medicaco AB, Sweden) (volume ratio of 20 mM NaOH and 10× PBS is 9:1)) to make the stock solution at a concentration of 50 µM ( = 1.7 mg/mL). ApoE protein solutions were aliquoted and kept at −80 °C. The dilution buffer alone was used in control experiments, termed the diluent group. All protein aliquots used for experiments experienced no more than two freeze-thaw cycles, to minimize variations in protein concentrations.

Lyophilized parental HS (GAG-HS01 BN1, Iduron, UK) with an average molecular weight of 40 kDa was gently mixed in milli-Q water (Millipore integral system, Molsheim, France) overnight at 4 °C to produce the stock concentration of 25 µg/mL. Later, the HS sample was biotinylated at its reducing end by oxime oxidation reaction as described in ref. ^101^ and stored at −20 °C until use.

Lyophilized streptavidin (Sigma) was dissolved in milli-Q water at 5 mg/mL, aliquoted, and stored at −80 °C. Thawed aliquots were used within a week and kept at 4 °C till use.

### Viruses and virus purification

Herpes simplex virus 1 (HSV1) strain KOS (VR-1493; ATCC, Manassas, VA) was produced and purified in our lab by ultracentrifugation through sucrose gradients^31^. In brief, propagation started with MOI 0.01 infection of GMK cells. Viruses from both cells and medium were collected, concentrated, and loaded on sucrose gradients (consisting of 2 mL each of 50, 40, and 30% sucrose layers, w/v) for purification. Cell-associated viruses were released by three freeze-thaw cycles. Centrifugation was done at 20,200 rpm for 2 h, with a SW41 Ti rotor and Optima XPN-80 Ultracentrifuge (Beckman Coulter). After centrifugation, virus particles sediment at the interface between the 50% and 40% sucrose layers. After collection of the virus-containing fraction, viruses were aliquoted in sucrose for infection studies on cultured cells. For biophysical experiments, sucrose was exchanged with PBS by an extra round of high-speed centrifugation (48,384×*g*, 20 min at 4 °C). It is to be noted that the presence of extracellular vesicles in our virus preparation cannot be excluded^61,63^, and that ApoE may also bind to those, given their likely direct interaction with lipid membranes^79^. Importantly, the distributions of extracellular vesicles through our sucrose layers, probed by common extracellular-vesicle markers CD 9 and CD 63, demonstrated that the majority of extracellular vesicles was not collected in our virus stock (Supplementary Fig. 14). In brief, the distributions of HSV1 and extracellular vesicles were analysed as below: after centrifugation, samples loaded on top of the sucrose gradients were harvested as one fraction, termed as “Top S (solution)”. Samples in the sucrose gradients were fractioned from top to bottom (1 mL/fraction). Samples from each fraction were processed for SDS-PAGE and western blot for detection of HSV1-gC (B1C1B4, diluted in 5% milk in PBS-tween)^30^, CD 9 (NBP2-67310, NOVUS, diluted in 2.5% BSA (bovine serum albumin, Merck) in PBS-tween), and CD 63 (NBP2-68077, NOVUS, diluted in 2.5% BSA in PBS-tween). Samples for SDS-PAGE and western blot were lysed in lysis buffer (0.05 M Tris-HCl pH = 8, 0.15 M NaCl, 1% Triton X-100, in H_2_O + protease inhibitor cocktails (Roche)) for 15 min on ice, then mixed with 6x laemmli buffer and boiled at 95 °C for 10 min. Boiled samples were quickly cooled down on ice and used for SDS-PAGE.

### Quantification of cell-associated virus

To quantify the total amount of cell-associated viruses, infected cells were harvested separately from the medium for DNA extraction and subsequent qPCR quantification of viral genome copies. DNA extraction was done according to the manufacturer’s instructions (Invisob Spin Virus DNA Mini Kit, IBL). Virus DNA was quantified by qPCR with primers targeting the US5 (unique short 5)^102^ gene as previously described^32^. A standard curve established with pI18-HSV1-US5 as the template was used to calculate the absolute quantities of HSV1 copies. The two primers used for qPCR were: HSV1-US5-F, (5′-GGCCTGGCTATCCGGAGA-3′); HSV1-US5-R (5′-GCGCAGAGACATCGCGA-3′). The probe for qPCR was 5′-6FAM-CAGCACACGACTTGGCGTTCTGTGT-Dark Quencher-3′. The qPCR program runs at 95 °C, 3 min; 40 cycles (95 °C, 15 s; 60 °C, 30 s). To specifically quantify virus particles on the cell surface, viruses were released by trypsin digestion and separated from cells by centrifugation. Experiments were carried out in 12-well plates. At indicated time points after infection, medium samples were harvested for qPCR or titration by plaque assay. Cells were then washed with PBS twice, followed by trypsin addition. After trypsin digestion, cells were resuspended in PBS and collected for centrifugation at 1500 rpm for 5 min. After centrifugation, cells and supernatants were separated. Released viruses in supernatants and intracellular viruses were then processed for DNA extraction and genome quantification by qPCR.

### Cell proliferation test

Cell proliferation (Supplementary Fig. 3B, C) was evaluated by the WST-1-based colorimetric assay according to the manufacturer’s instructions (Roche, 5015944001). In brief, cells were seeded in 96-well plates and cultured overnight. On the second day, the medium was changed to 50 µL of new medium containing the designated ApoE, and the cells were further cultured for 24 h. Four hours prior to analysis, WST-1 reagent was added (5 µL/well), followed by absorbance measurement at 440 nm by an ELISA reader. The corresponding culture medium was included as a control, and this readout was subtracted from the experimental groups.

### Quantification of virus release rate from infected cells

The release rate of virus was investigated in the premise that similar amounts of viruses were found on the cell surface (Fig. 2B) in all groups at the indicated starting time point. The virus release into the medium (Fig. 2C, without refreshing of any ApoE) was then monitored for 1 or 2 h, after the removal of the supernatants and three times wash of cells with ice-cold PBS. Released viruses were later quantified by plaque assay.

### Purified HSV1 and ApoE 4 incubation, complex separation, and fluorescence labeling

Incubation and fluorescence labeling of purified HSV1 and ApoE 4 protein was done at the same time for the experiments described in Figs. 3–6. About 2 × 10^8^ PFU HSV1, 8 µL ApoE (50 µM in stock), and 1 µL fluorescent lipophilic dye 1,1′-dioctadecyl-6,6′- di(4-sulfophenyl)-3,3, 3′,3′-tetramethylindocarbocyanine (SP-DiIC18(3), or in short SP-DiI) (D7777, Invitrogen, 400 µM in stock) were mixed in PBS in a 1.5 mL Eppendorf tube with the final volume as 100 µL. The mixtures were incubated under rotation for 1 h at room temperature, followed by adding 100 µL/sample Capto-core beads (Capto Core 700, 17548101, Cytiva) to the same tube and a further 1 h incubation at 4 °C. Capto-core beads were separated by centrifugation at 800×*g*, 2 min. The supernatants were collected and further purified by running through S-200 columns (MicroSpin^TM^S-200 HR Columns, 27512001, Cytiva). In parallel, HSV1 mixed with diluent (without ApoE 4 added), was included as a control for comparison in the designated experiments. Labeled HSV1 with ApoE or diluent was kept on ice for a few hours (3–4 h) before adding to SLBs for experiments (Fig. 3). When HSV1 without ApoE incubation was used for kinetic analysis (Figs. 7), 2 × 10^8^ PFU virus was freshly labeled prior to each experiment with 1 µL of fluorescent lipophilic dye 3,3′-dioctadecyl-5,5′-di(4-sulfophenyl) oxacarbocyanine, sodium salt (SP-DiOC18 (3), in short SP-DiO) (D7778, Invitrogen, 400 µM in stock), followed by size exclusion and buffer exchange by filtration through MicroSpin S-200 HR columns (27-5120-01, Cytiva)^29,65^. After labeling, the amount of lost viruses was estimated to 60% using a Förster Resonance Energy Transfer (FRET)-based assay^103^.

### Virus attachment kinetics to cells

Virus-binding quantification of HSV1 and ApoE 4-coated HSV1 (Fig. 4) was done in 12-well plates by qPCR. To get the same input, HSV1 and ApoE 4-coated HSV1 prepared as described above (with purified HSV1 via ultracentrifugation, no freeze-thaw cycle) were quantified by qPCR after DNA extraction (as described above). After quantification, 50,000 copies of each were diluted in 150 µL infection medium (DMEM, 1% FBS, 20 mM HEPES, and 1% Penicillin and Streptomycin) and added to cells for binding synchronization on ice while rocking the plate every 10 min. After the selected periods of binding time (*t* = 15, 30, 45, 60, 90, and 120 min) on ice, unbound viruses in the solutions were removed, and cells were quickly washed with ice-cold PBS twice. The attached viruses were harvested together with cells for DNA extraction and quantification by qPCR.

Virus binding in Supplementary Fig. 13B. was quantified by qPCR as described above in 12-well plates. Prior to adding viruses, cells were treated with ApoE for 4 h. About 5000 PFUs and 25,000 PFUs/well of virus were added for attachment on ice. Quantification of different PFUs as inputs by qPCR revealed an almost perfect linear regression^32^, confirming that this approach was viable.

### Viral entry kinetic and entry efficiency

Virus entry efficiencies in Fig. 4B were investigated by a previously described method^32^. Before the entry assay, virus binding was done in the same way as in binding quantification. Based on our previous titration results of HSV1 and ApoE 4-coated HSV1, the inputs were estimated as 200 PFUs for both forms of HSV1. Thereafter, viruses attached to the cell surface were either inactivated immediately (*t* = 0 min) by low pH buffer (pH = 3, 40 mM citric acid, 10 mM KCl, 135 mM NaCl)^58,104^ or shifted to 37 °C for active entry for selected periods of times (*t* = 15, 30, 60, 90, and 120 min) before low pH buffer inactivation. Low pH inactivation was done by adding 350 µL/well of low pH buffer for 2 min on ice, shaking every 20–30 s, followed by two PBS washes. After inactivation of the uninternalized virus, cells were covered by 1% agarose in DMEM (5% FBS) for another 3 days culture until obvious plaque formations. A low MOI was chosen to ensure a clear readout of plaque formation. Cells were then fixed with 4% formaldehyde (in PBS) and stained with crystal violet (1% crystal violet in 20% ethanol solution). The number of plaques was then counted for each condition.

When comparing the entry efficiencies of HSV1 and ApoE 4-coated HSV1, the different binding efficiencies of the two virus forms (Fig. 4A) were considered by quantifying the actual amounts of attached viral particles from a parallel group to the experimental groups after 1 h synchronization on ice via qPCR. From three independent experimental repeats, the ratios of (HSV1 + ApoE 4)/HSV1 attached to the cell surface were measured as 0.83, 0.59, and 0.75. The normalization of HSV1+ApoE 4 entry was done by dividing the counted plaque numbers by the corresponding ratios (Fig. 4B).

Virus entry kinetics and efficiency in Supplementary Fig. 13C, D were analysed in a similar way. In brief, a low MOI (200 PFU) was chosen as the input for all the groups. The normalization of the virus entry was then done by dividing the number of plaques by their corresponding binding factors (binding ratios normalized to the diluent group, Supplementary Fig. 13B).

### Small unilamellar vesicles for (native) supported lipid bilayers

1-palmitoyl-2-oleoyl-glycero-3-phosphocholine (POPC) (850457P), 1,2-dioleoyl-*sn*-glycero-3-[(*N*-(5-amino-1-carboxypentyl) iminodiacetic acid) succinyl] (18:1 DGS-NTA(Ni)) (790528 P), 18:1 Biotinyl Cap PE (DOPE-Cap-β) (870273 P), *N*-palmitoyl-sphingosine-1-{succinyl[methoxy(polyethylene glycol)5000]} (PEG) (880280 P), and (1,2-dioleoyl-*sn*-glycero-3-phosphoethanolamine-*N*-(7-nitro-2-1,3-benzoxadiazol-4-yl) (NBD-PE) (810145) were purchased from Avanti Polar Lipids (Alabaster, AL, USA). 1,2-dihexadecanoyl-*sn*-glycero-3-3-phosphoethanolamine, triethylammonium salt (TxRed) was purchased from Thermo Fisher Scientific (T1395MP). Small unilamellar vesicles (SUVs) were prepared by extrusion using a mini extruder equipped with a 1 mL syringe (610020 and 610017; Avanti Polar Lipids) as described previously^29^. Pure POPC vesicles, POPC:PEG vesicles (99.5:0.5, molar ratio), and POPC:TxRed vesicles (99:1, molar ratio) were all prepared in PBS by extrusion through a 100 nm polycarbonate membrane at least 11 times. POPC:TxRed was prepared at a stock concentration of 4 mg/mL, the others at 1 mg/mL. POPC:DGS-NTA vesicles (96:4, molar ratio) and POPC:DOPE-Cap-β (90:10, molar ratio) were prepared in HEPES saline buffer (HBS, 10 mM HEPES and 150 mM NaCl; pH 7.5) and PBS, respectively at a stock concentration of 1 mg/mL by extrusion through a 50 nm polycarbonate membrane at least 21 times and were stored under N_2_. All stocks were stored at 4 °C.

### Binding assay of HSV1 to surface-immobilized His-tagged ApoE

Borosilicate glass cover slides with a diameter of 22 mm (631-0158P, round, No.1, VWR) were cleaned using 7x detergent (MP Biomedicals, CA) and milli-Q water with 1:6 (volume ratio) solution close to boil for 2 h, rinsed extensively and stored in milli-Q water. Before use, the slides were rinsed with milli-Q water, N_2_ dried, and treated in a UV/ozone (UV Ozone Cleaner -ProCleaner™ Plus, Bioforce, IA, USA) for 30 min.

Supported lipid bilayers (SLBs) were formed from SUVs by the method of vesicles spreading, through 30 min exposure of 50 µg/mL vesicle solution in HBS supplemented with 10 mM NiCl_2_ to freshly cleaned glass coverslip. The coverslip was fixed onto a custom Teflon holder using a bi-component Twinsil glue (Picodent, Germany), creating eight wells of equal volume. All incubation steps were performed in a still solution. The excessive SUV material was removed from each well by rinsing 20 times with 100 µL PBS. After rinsing, SLBs were incubated with POPC vesicles for 30 min at a final concentration of 37.5 µg/mL to ensure the formation of a good quality bilayer by filling up any potential defects. After rinsing in PBS, three wells were exposed to His-tagged ApoE 4 at a final concentration of 100 µg/mL for 30 min. A control experiment was also run with denatured ApoE. For denaturing His-ApoE 4, the protein (200 µg/mL) was diluted in 4 M denaturant guanidine hydrochloride and boiled at 80 °C for 15 min prior to incubation using similar conditions as for the non-denatured ApoE: Wells that were not exposed to His-tagged ApoE 4 were used as control. After rinsing with PBS, six wells were exposed for 30 min to fluorescently labeled HSV1 particles with or without preincubation with ApoE 4. Here, 2 × 10^8^ PFU of HSV1 purified via ultracentrifugation, 4 µM ApoE, and 40 µM fluorescent lipophilic dye (SP-DiI) were incubated for 1 h in PBS and separated (see purified HSV1 and ApoE incubation section for details). In a control experiment, ApoE antibodies (Invitrogen, PA5-27088) were included to block HSV1-ApoE 4 interactions at the final concentration of 150 µg/mL for 1 h incubation. Images (704 × 704 pixels with a 0.183 µm pixel width) of bound particles at six or twelve randomly selected positions were collected using TIRFM with an inverted Nikon (Japan) Eclipse Ti-E2 microscope, an oil immersion 60X objective (Nikon, NA: 1.49) and a 561 nm laser. The images were analysed using ImageJ. A Gaussian blur (sigma:1 pixel) was applied to all images to reduce the noise and particles were counted after thresholding. Raw particle numbers were normalized between samples to account for differences in the stock concentration. Experiments were repeated twice.

To confirm the interaction between ApoE 4 and HSV1, silica beads (SiO2-R-3.0, microParticles GmbH, Berlin, Germany), 50 µl per sample, were washed three times in HBS and incubated in 50 µl of Ni-NTA liposome solution (100 µg/ml 95:5 POPC:DGS-NTA liposomes, 2.5 µg/ml of 99:1 POPC:NBD-PE and 20 mM NiCl2 in HBS) for 30 min. The beads were then incubated in 20 µg/ml of His-tagged ApoE 4 or protein G (RPG-S3140, ACROBiosystems, Newark, DE, USA) in HBS or only HBS and then washed three times in 0.2% BSA solution in HBS (blocking solution). Finally, the beads were incubated with 25 µl of SP-DiI-stained HSV1 (see above) in a blocking solution. After virus removal, the beads were resuspended in 100 µl of HBS per sample (treated bead solution) and used for qPCR and flow cytometry analysis. All incubations were performed at room temperature and under rotation to prevent bead sedimentation. Samples were washed three times in 500 µl of HBS between each step when not otherwise stated.

Flow cytometry was used to determine the beads density in each sample to normalize the loading for qPCR and western blot. About 5 µl of treated beads solution was diluted in 200 µl of HBS and measured with a ZE5 cell Analyser (Bio-Rad). NBD and SP-DiI signals were detected to confirm the presence of the lipid bilayer on beads and the capture of virions and extracellular vesicles stained with the lipophilic dye. About 2 µl of untreated silica beads diluted in 500 µl of HSB were used as negative control. Flow cytometry data were analyzed using FlowJo (BD, Franklin Lakes, NJ, USA).

For qPCR analysis, 45 µl of treated beads solution was diluted to a final volume of 200 µl in HBS and processed for DNA extraction using Invisob Spin Virus DNA Mini Kit (IBL). The numbers of HSV1 genome copies were quantified by qPCR as described above in the section for quantification of cell-associated virus.

### Determination of the relative concentrations of fluorescently labeled HSV1 samples

The concentration of labeled particles in each sample was calculated using an assay referred to as “bouncing particle analysis”. In brief, this method measures the number of particles that transiently diffuse close to the glass surface into the TIRF excitation volume without interacting with the substrate, i.e., “bouncing” off the surface. Since particles randomly diffuse in the solution, the number of particles detected during this procedure is linearly proportional to the particle concentration in the solution. The measurement was performed on pure POPC SLBs, which are resistant to HSV1 attachment, to minimize the interaction between the particle and the substrate. The timelapses were recorded in TIRF mode with the 60X oil immersion objective using a 561 nm laser at 50% power and at 10 fps for 1 min. The movies were analysed using an in-house MATLAB (MathWorks, USA) script using the same principle as described for the kinetic analysis (see section Virus binding kinetics). Only particles detected on the surface for less than 10 frames or 1 s were considered non-interacting with the substrate and counted as “bouncing particle” events. The cumulative sum of new bouncing particles was then calculated versus time and fit to determine the rate of arrival (slope of the cumulative sum). This factor is linearly proportional to the particle concentration in the solution and was used to normalize the concentrations across samples.

For each sample, movies were recorded at three different randomly selected positions, analysed, and averaged to extract the arrival rate.

### Immunostaining of HSV1 particles for colocalization experiments

To carry out colocalization experiments (Supplementary Fig. 5), round glass coverslips were cleaned, UV-treated, and glued on Teflon or PDMS holders. HSV1 particles were stained with the lipophilic dye SP-DiI as described above. The virus solution was diluted 1:40 in PBS and incubated onto the coverslip for 20 min to ensure particle adsorption onto the glass surface. After washing in PBS, the surface was passivated using 1% BSA in PBS for 1 h. The virions were immunostained using a cocktail of anti-HSV1-gB, gC, gD, and gE glycoproteins^30^ at a concentration of 1:100 in 1% BSA for 1 h, followed, after thorough washing, by Alexa-488-conjugated anti-mouse IgG antibody (A-21202, Thermo Fisher) staining at a concentration of 1:200 in 1% BSA for 1 h. For colocalization of the capsid protein (VP5) with the membrane of HSV1 particles (Supplementary Fig. 5C), SP-Dil stained particles were immobilized on a clean glass coverslip mounted in a Teflon or PDMS holder as described earlier. After washing with PBS, HSV1 particles were either fixed for 10 min in 2% w/v formaldehyde or incubated in PBS and then treated with 1% SDS (sodium dodecyl sulfate) to permeabilize the viral envelope for 15 min. The wells were extensively washed with PBS and passivated with 2% BSA in PBS (blocking solution) for 1 h. The virions were immunostained with a mouse monoclonal IgG against VP5 (6F10, sc-13525, Santa Cruz Biotechnology, USA) at the concentration of 1:50 in a blocking solution for 1 h. After thorough rinsing with PBS, the wells were exposed to a secondary antibody (alexa-488-conjugated anti-mouse IgG antibody) at a concentration of 1:200 in blocking solution for 30 mins. After rinsing with PBS, images were collected and analysed as described above. All VP5 staining experiments were carried out at 37 °C unless otherwise stated and repeated twice. Negative controls were performed by incubating with PBS instead of the permeabilization step or instead of incubation with the primary antibody. After rinsing with PBS to remove the unreacted antibody, images of the adsorbed particles were collected using TIRFM at 488 and 561 nm lasers. The images were analysed with a custom MATLAB script to detect fluorescence spots in both wavelengths and determine the fraction of colocalized particles, i.e., particles stained by the lipophilic dye and anti-HSV1 antibodies which present viral glycoproteins. Particles were detected using the 2D peak find algorithm for EFA using a fixed threshold on the peak prominence to separate the particles from the background noise. Peaks detected in the two channels were considered colocalized if the distance between their centers was less than 2 pixels (372 nm).

### Native supported bilayer preparation

Native membrane vesicles (NMVs) were prepared according to a protocol adapted from ref. ^64^. Confluent cells were rinsed with cold PBS, and then harvested on ice in ice-cold harvest buffer (PBS mixed with EDTA-free protease inhibitor cocktail, Roche) using a cell scraper. Harvested cells were pelleted via centrifugation at 600×*g* for 10 min, before they were disrupted by four passes through a CF1 continuous flow cell disruptor (Constant Systems, U.K.) set at 2500 psi. The suspension was then centrifuged at 2000×*g* for 10 min to pellet nuclei and larger organelles, and then at 6000×*g* for 20 min to pellet mitochondria. Finally, the suspension was centrifuged at 150,000×*g* for 90 min to pellet the NMVs. NMV pellets were resuspended in harvest buffer and purified via sucrose gradient, to separate plasma membrane vesicles from organelle membrane vesicles. The suspension was mixed with an equal volume of 80% sucrose solution, and then layered with a 30 and 5% sucrose solution, respectively. All sucrose solutions were made with harvest buffer. The sucrose cushion was then centrifuged at 273,000×*g* for 2.5 h. The NMVs form a hazy band at the 5 and 30% sucrose interface, which was harvested, flash-frozen, and stored at −80 °C. All centrifugations were done at 4 °C. Concentration of NMV material was measured using a FRET-based assay described previously^103^.

To form the nSLBs, NMVs were mixed with synthetic POPC:PEG vesicles in a 15:85% ratio, according to the surface area determined with the FRET assay^103^. Mixing was done in 1.5 mL microcentrifuge tubes to a final concentration of 200 µg/mL and a final volume of 60 µL. The mixture was sonicated for 20 min at 40 °C in a bath sonicator (37 kHz, Elmasonic S40H, Germany). After sonication, vesicles were then mixed with 0.3 µL of 0.5 µg/mL POPC:TxRed vesicles.

Borosilicate glass cover slides with a diameter of 24 mm (631-0161, round, No.1, VWR) were cleaned using 7x as described above, but without UV-ozone treatment. Bilayers were formed in custom-made 10 µL PDMS wells attached to the cover slides. About 10 µL of vesicle mixture was injected into the wells and incubated for 30 min at 37 °C. Afterward, wells were rinsed 10x with 10 µL PBS, before passivating the bilayer with POPC vesicles at a final concentration of 50 µg/mL. Bilayers were then visually inspected for unruptured POPC:TxRed vesicles to confirm bilayer formation before proceeding.

### Surface immobilization of heparan sulfate

For TIRF microscopy-based kinetic experiments with surface-bound HS films, round glass coverslips were cleaned, UV-treated, and glued on a Teflon holder as described above. SLBs containing 10% biotinylated lipids (POPC:DOPE-Cap-β (90:10, molar ratio)) were formed at a final concentration of 100 µg/mL. After SLB formation, each well was rinsed 14x with 150 µL PBS and then incubated with pure POPC vesicles at 50 µg/mL for 30 min to get good-quality SLBs. After the PBS rinse, each well was incubated with streptavidin in PBS for 30 min at a final concentration of 50 µg/mL. After rinsing, each well was incubated with the final 2% v/w formaldehyde for 15 min to crosslink and prevent lateral diffusion of streptavidin on the SLBs. Following PBS rinse, each well was incubated with a final concentration of 10 µg/mL biotinylated HS (b-HS) for 30 min. After rinsing, all wells were incubated with 50 µg/mL pure POPC vesicles for ~1 h before incubating with virus samples. Wells were kept in this solution and to further reduce non-specific binding, virus samples were diluted in the same solution (i.e., 50 µg/mL pure POPC vesicles in PBS).

### Equilibrium fluctuation analysis

Kinetic timelapses were imaged with the Nikon Ti-E2 microscope in TIRF mode using a 60X oil immersion objective and a 561 nm laser. nSLBs or HS films were incubated with fluorescently labeled virus particles for 1 h before recording kinetics at either 15 s/frame for 20 min for 80 frames (data in Fig. 5B), 90 min for 361 frames (data in Fig. 5C), 1 s/frame for 20 min for 1201 frames (data in Fig. 6) or 30 s/frame for 180 min for 361 frames (data in Fig. 7). Timelapse field-of-view is 704 × 704 pixels with a resolution of 0.183 µm/pixel.

Movies were stabilized using an in-house MATLAB script. The MATLAB script takes as reference image the first frame and performs an image registration based on maximizing the cross correlation between the current and reference frame. The reference frame is updated after one third of the length of the movie to improve the registration of rapidly changing videos. In the case a shift larger than 15 pixels in any direction is detected, the script updates the reference frame to the last analysed frame and repeats the registration. If no improvement is observed, a more computationally intensive multimodal registration algorithm is attempted. In the case of low density of particles, the script averages over all the frames and crops the field of view to only consider the area presenting the ten brightest spots to reduce possible errors in the correlation calculation due to random noise. Only rigid translation is considered in every case.

We then performed EFA of the stabilized movies using an in-house MATLAB script with improved detection capabilities from what was previously described^29^. In brief, in each frame, particles are detected by performing two successive 2D peak scans. Firstly, peaks of prominence higher than a threshold set by the user were identified as particles. The threshold was selected to minimize the number of detected particles that do not carry viral glycoproteins, as described in the Supporting information. A 40% lower threshold was applied for particles already detected in previous frames to minimize the effects of bleaching. The same thresholds were used for all samples in the same experiments. The nearest neighbor algorithm was then used for linking the particle by comparing successive frames with a maximum linking distance of 2 pixels. Possible errors in linking causing the fragmentation of single tracks, thus false attachment, and detachment events, were corrected by comparing the position of tracks ending and beginning in successive frames and linking them if within 1 pixel. Finally, detachment events causing an intensity drop lower than three times the standard deviation of the background were discarded.

For the kinetic analysis, a particle was considered bound if it remained on the surface for at least three consecutive frames. The particle arrival rate was determined by fitting the plot displaying the number of newly detected particles over time with a linear fit (y = Ax + B). To ignore artifacts in the analysis of the initial binding events, the first 13% of data points were excluded from the fitting. The particle dissociation rate was quantified by plotting the number of bound particles as a function of the residence time on the surface and fitting the curve with a double exponential function decay with an offset ($f\left(t\right)=\,{{\rm{A}}}_{1}\cdot \exp \left(-{{k}_{{\rm{off}}}}^{1}\cdot t\right)+\,{{\rm{A}}}_{2}\cdot \exp \left(-{{k}_{{\rm{off}}}}^{2}\cdot t\right)+\,{y}_{0}$), where A_1_ and A_2_ = amplitude and ${y}_{0}$ = offset to extract two dissociation rates and irreversible fraction. The irreversible fraction was calculated using the following expression: $\frac{{y}_{0}}{{(y}_{0}+{A}_{1}+{A}_{2})}$. Only particles that bind during the first half of the timelapse were included in the analysis, to avoid biasing the data towards shorter residence times. All binding experiments were normalized to a control which was the binding of naked HSV1 to nSLBs (Fig. 5), to HS films (Fig. 6), or to nSLBs from HEK cells without the induction of ApoE 4 (HEK-) (Fig. 7). Each data point was normalized to its respective control from the same experiment to account for day-to-day variability. After normalization, the results were presented as relative values for association and dissociation rates, and irreversible fraction.

### Equilibrium fluctuation analysis data thresholding

To minimize the signal from fluorescent particles not carrying viral glycoproteins, in EFA experiments the threshold used for particle detection was carefully selected using the following procedure. Firstly, in the colocalization experiments, the detection threshold of the 561 nm channel (lipophilic dye labeling) was varied from a level just above the background noise, to one that allowed the detection of only the brightest spots. Both the number of detected particles and the percentage of particles colocalizing with the Ab signal were recoded (dark blue and red circles in Supplementary Fig. 5D). The percentage of colocalised particles initially increases with the threshold level before reaching a plateau. The onset of the plateau was chosen as the optimal threshold level (${T}_{{\rm{col}}}^{{\rm{opt}}}$), maximizing the number of detected particles and the percentage of particles carrying viral glycoproteins. Secondly, since the acquisition of EFA movies limits the exposure time and intensity of the exciting laser, the threshold level needs to be adjusted from ${T}_{{\rm{col}}}^{{\rm{opt}}}$. A similar threshold scouting algorithm was applied to selected images from EFA timelapses to determine the relation between the number of detected particles and threshold level. The resulting curve was matched to the one observed in the colocalization experiment by applying a conversion factor ($c$) to the threshold values (light blue diamonds in Supplementary Fig. 5D). ${T}_{{\rm{col}}}^{{\rm{opt}}}$ was divided by $c$ to find the equivalent threshold level in the acquisition conditions used in EFA experiments, ${T}_{{\rm{EFA}}}^{{\rm{opt}}}={T}_{{\rm{col}}}^{{\rm{opt}}}/c$.

### HSV1 binding to immobilized ApoE isoforms

To measure the binding kinetics of HSV1 to surface-immobilized ApoE isoforms (Supplementary Fig. 7), coverslips were cleaned in a boiling solution of 1:10 7x detergent in Milli-Q and UV-treated. About 10 µl PDMS wells were used as described above for nSLB preparation. The SLBs containing 5% NTA-95% POPC were formed by spontaneous rupture of 50 nm vesicles with the same composition at a final concentration of 100 µg/mL in HBS supplemented by 10 mM NiCl_2_. After rinsing, the bilayer was exposed to His-tagged ApoE isoforms (2, 3, and 4) at a concentration of 100 µg/ml for 1 h. No protein was added for the negative control. Simultaneously, HSV1 was fluorescently labeled as described above. After rinsing the surface in PBS, 5 µl of virus solution was added to 5 µl of PBS remaining in the well and incubated for 1 h to reach equilibrium. The samples were imaged in TIRFM as described above at 20 s/frame for 1.5 h. All rinsing steps were performed by adding 10 µl of buffer to a sample volume of 5 µl at least seven times.

The videos were analysed using the EFA method described above. A single exponential fit with offset was used to determine the multivalent dissociation rate constant (${k}_{{\rm{off}}}$):

$$y=A\exp \left(-{k}_{{\rm{off}}}t\right)+{\rm{IF}}$$

where IF represents the fraction of particles that behave as irreversibly bond to the substrate in the timescale of the experiment, *A* is a fitting constant and *t* is the elapsed time since particle attachment. The normalized dissociation constant (${{\rm{K}}}_{{\rm{D}}})$ was determined by dividing ${k}_{{\rm{off}}}$ by the association rate for each isoform and by normalizing the result by the average value obtained for ApoE 2.

Equal loading of the ApoE isoforms onto the NTA-presenting bilayer and the stability of the resulting bond were confirmed prior to the TIRFM assay using quartz crystal microbalance with dissipation monitoring (Supplementary Fig. 7D). QCM-D sensors (5 kHz fundamental frequency, silica coating AWS SNS 000049A from advanced wave sensors (AWSensors), Spain) were cleaned with 2% SDS for 1 h, thoroughly rinsed with milli-Q water, dried with N2, and treated in a UV/ozone cleaner for 30 min. The QCM-D cell and tubing were cleaned in Cobas detergent (Roche, Switzerland) for 1 h, then thoroughly rinsed in milli-Q water and N2 dried. Experiments to follow the binding of His-tagged ApoE isoforms on NTA bilayers were carried out using an X4 system (AWSensors, Spain) with four channels, a flow rate of 20 µL/min, and a working temperature of 22 °C in PBS buffer which was filtered with 0.2 µm filters (Sarstedt, Germany), and degassed in a sonicator (Elmasonic, S40H, Singen, Germany). Frequency (Δf) and dissipation (ΔD) shifts were recorded at six overtones (3, 5, 7, 9, 11, 13), with data for *i* = 3 presented in Supplementary Fig. 7D.

### Co-immunoprecipitation

Huh-7 cells, expressing ApoE 3, were used for interaction studies between ApoE and HSV1 glycoproteins (see Supplementary Fig. 12). Cells in T75 flasks were mock treated or infected with HSV1 at MOI 10. At the indicated times, flasks were placed on ice and washed with ice-cold PBS once. Cells were lysed by adding 1 mL lysis buffer (0.05 M Tris-HCl pH = 8, 0.15 M NaCl, 1%Triton X-100, in H_2_O + protease inhibitor cocktails (Roche)) to each flask and collected into Eppendorf tubes. The lysis was done by incubating samples on ice for 20 min. Cell debris was separated via centrifugation at 13,000 rpm for 10 min at 4 °C. After centrifugation, 60 µl of cell lysates were kept for input detections. The rest of the lysates were used for co-immunoprecipitation with anti-ApoE (Invitrogen, PA5-27088) or anti-HSV1-gE antibodies.

Co-immunoprecipitation was done with 30% protein A agarose beads (Sigma, P3476-5), 50 µl/sample. Before incubation, beads were washed three times in 1 mL lysis buffer via centrifugation (3000 rpm, 3 min at 4 °C). The washed beads (without adding antibodies) were incubated with cell lysates for 2 h at 4 °C to clear unspecific binding. The cleared cell lysates were incubated with 3 µg of ApoE or HSV1-gE antibodies at 4 °C overnight. On the following day, new agarose A beads were washed and added to the lysates + antibody solutions for another 90 min incubation at 4 °C. Then, the complexes (beads, antibodies, and the attached proteins/protein complexes) were collected and washed with lysis buffer 5 times by centrifugation at 3000 rpm, 3 min, and 4 °C. After the last wash, the lysis buffer was removed with a syringe, and 60 µl 2X Laemmli buffer/sample was added and proceeded for SDS-PAGE and western blot. Antibodies were used to probe ApoE, HSV1-gB (1B11D8), HSV1-gC (B1C1B4), HSV1-gD (C4D5G2), HSV1-gE (B1E6A5)^30^, or tubulin (Invitrogen, MA5-16308-HRP).

## Supplementary information

Supplementary information-Liu et al

## Data Availability

The datasets used and/or analysed during the current study are available from the corresponding author on reasonable request.

## Code availability

The underlying code for data analysis of this study is not publicly available but may be made available to qualified researchers on reasonable request from the corresponding author.
