# Supplementary material for: Recruitment of apolipoprotein E facilitates Herpes simplex virus 1 attachment and release
Source: Npj Viruses. 2025 Feb 22;3:13. doi: 10.1038/s44298-025-00099-9 (PMC11846946; doi:10.1038/s44298-025-00099-9)
Supplement: Supplementary file 1 — Supplementary information-Liu et al [file 44298_2025_99_MOESM1_ESM.pdf]

Supplementary Information

Recruitment of apolipoprotein E facilitates Herpes simplex virus 1 attachment, entry, and release

Lifeng Liu[a,b,c#], Fouzia Bano[a,b,c†], Dario Valter Conca[a,b,c†], Konrad Thorsteinsson[a,b,c†], Sanduni Wasana Jayaweera[a], Damien Avinens[a,b,c], Hudson Pace[a,b,c], Hugo Lövheim[b,d], Anders Olofsson[a], Marta Bally[a,b,c#]

[a]Department of Clinical Microbiology, Umeå University, Sweden.

[b]Wallenberg Centre for Molecular Medicine, Umeå University, Sweden.

[c]Umeå Centre for Microbial Research, Umeå University, Sweden

[d]Department of Community Medicine and Rehabilitation, Umeå University, Sweden.

[†] These authors contribute equally to this work.

[#]Correspondence

Lifeng Liu, lifeng.liu@umu.se; Marta Bally, marta.bally@umu.se. Department of Clinical Microbiology, Umeå University, Sweden.

Supplementary figures and table

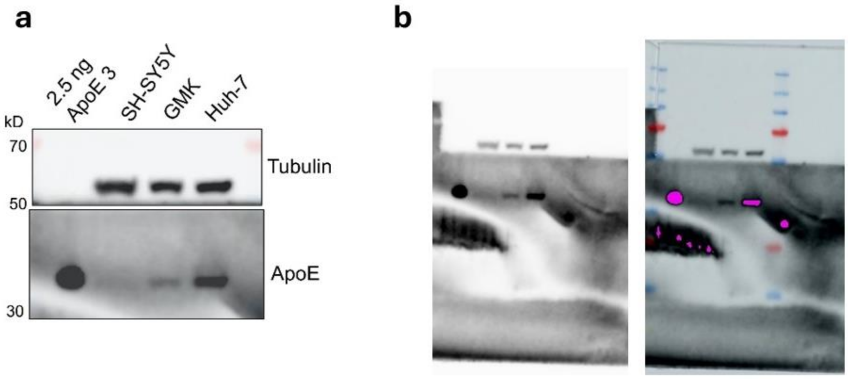

Supplementary Figure 1: ApoE expression levels in different cell lines. (**a**) Same amounts of SH-SY5Y, GMK, or Huh-7 cells were lysed and analysed by western blot for ApoE expression. Tubulin and ApoE were probed. (**b**) Original blots with (right) or without (left) a protein ladder are presented.

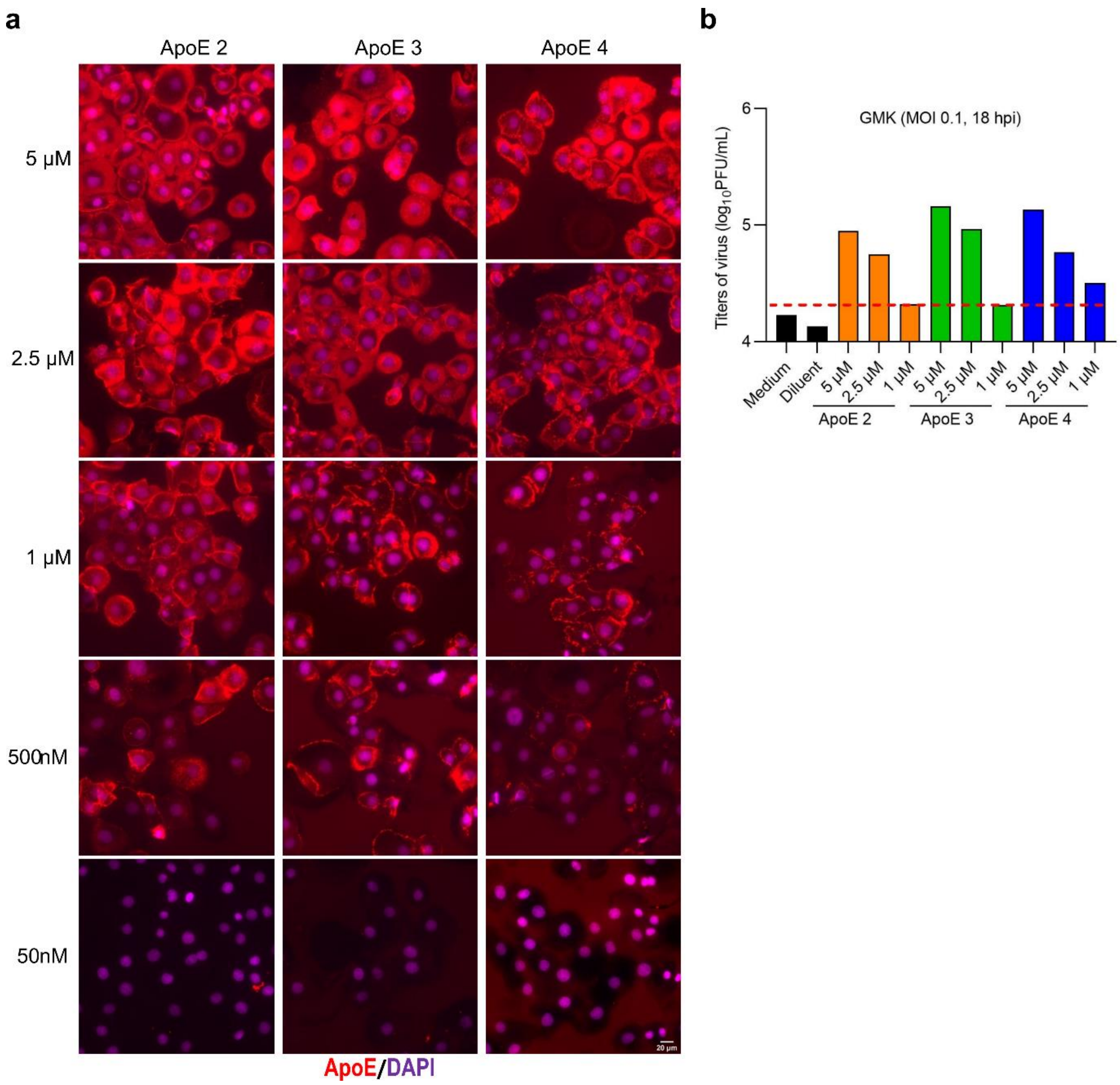

Supplementary Figure 2: **ApoE uptake and HSV1 infection in GMK cells with different ApoE concentrations (a)** ApoE 2, 3, or 4 was diluted in normal culture medium and added to GMK cells in a 24-well plate with indicated concentrations. After 8 h incubation, cells were fixed with 4% formaldehyde for 15 min and permeabilized with the permeabilization buffer (0.5% Triton X-100, 20 mM glycine in PBS) for 15 min. The fixed samples were incubated with a blocking buffer for 30 min, followed by incubation of primary antibody against ApoE (PA5-27088 ThermoFisher Scientific, 1:1000 in blocking buffer) for 1h and secondary antibody (A32733 ThermoFisher Scientific, Alexa Fluor™ Plus 647, 1:500), together with DAPI staining, for 1 h. All the procedures were carried out at room temperature. Images were taken with Nikon (Japan) Eclipse Ti-E2 microscope, 60X oil objective. Representative images were shown for each group. **(b)** HSV1 growth 18 hpi was analysed by plaque assay with indicated concentrations of ApoE 2, 3, or 4 added at 1 hpi. Results were generated from a single experiment.

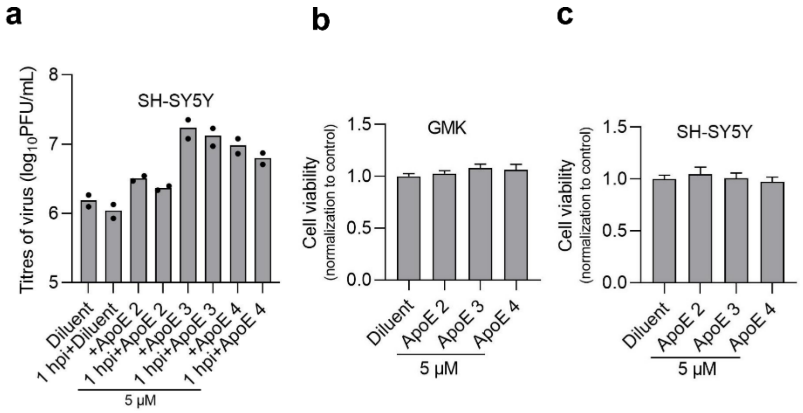

Supplementary Figure 3: HSV1 infection on SH-SY5Y pretreated with ApoE and cell toxicity test after adding ApoE to cells. (a) HSV1 growth was analysed by plaque assay after infection of SH-SY5Y at MOI 0.1, 24 hpi, where 5 µM ApoE was either pre-incubated overnight (labelled as +ApoE) or added after 1 h virus inoculation (labelled as 1 hpi + ApoE). GMK **(b)** or SH-SY5Y **(c)** cell growth was analysed in the presence of 5 µM ApoE isoforms. The data were normalized to the average absorbance values of the diluent group.

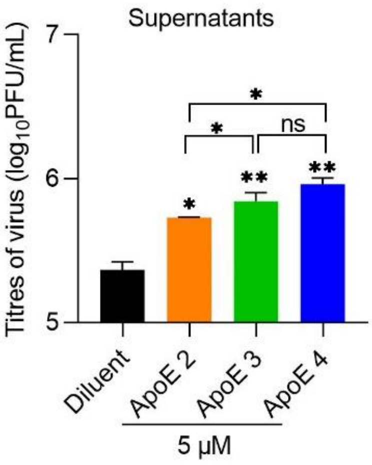

Supplementary Figure 4: HSV1 in the supernatants titrated by plaque assay. GMK cells were infected with HSV1 (MOI 0.1) and ApoE or diluent was added at 1 hpi. At 20 hpi, supernatants and cell samples were separated. Viral genomes in the supernatants (the released HSV1) were quantified by qPCR.

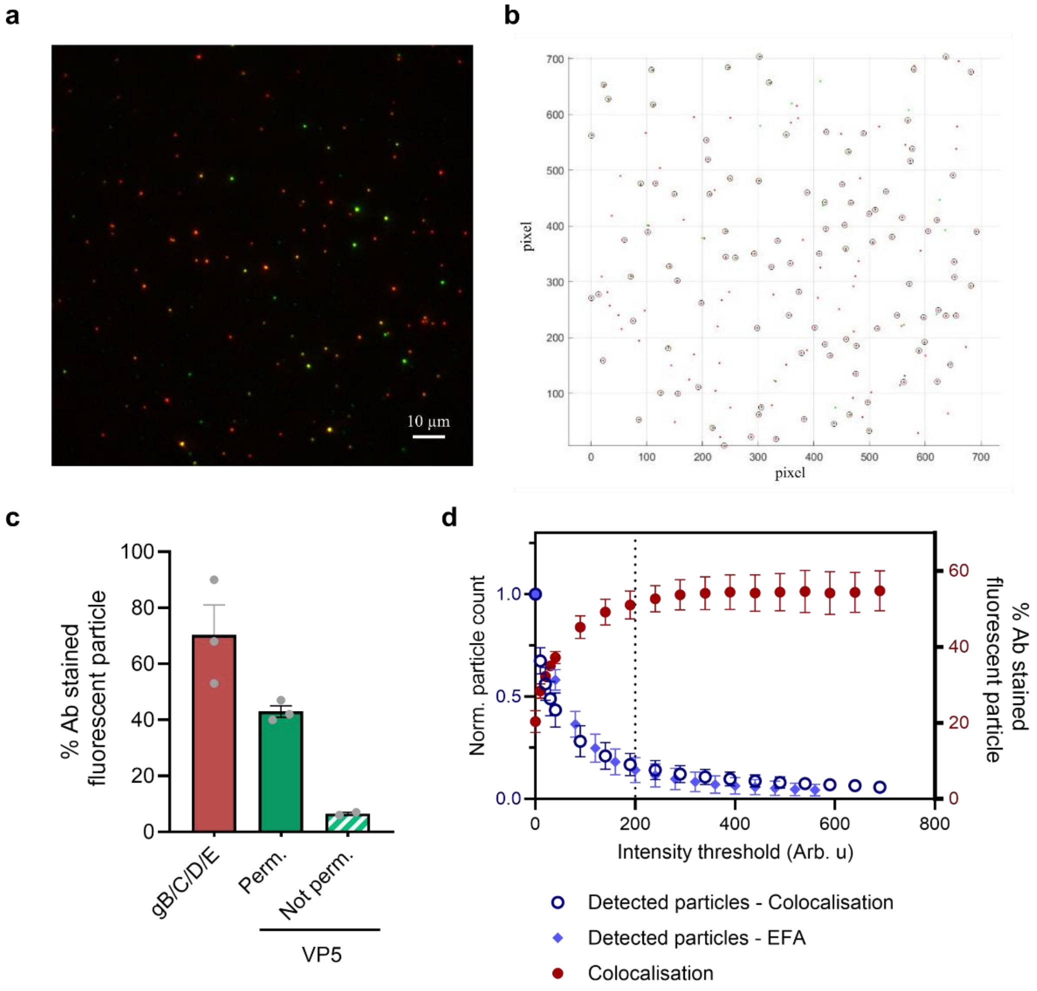

Supplementary Figure 5: Colocalization experiments to identify extracellular vesicles and thresholding strategy for data analysis. (a) An example TIRFM image of HSV1 particles adsorbed on a glass surface and double stained with lipophilic dye SP-DiI (red) and a cocktail of antibodies against viral glycoproteins gB, gC, gD, and gE (green). No antibody signal was detected in control experiments carried out in the absence of primary antibodies. **(b)** Result of the colocalization analysis for the image in (a) displays the detected particles in the 488 nm channel (green dots), 561 nm channel (red dots), and the particles colocalising (black circles). **(c)** Percentage of double stained particles at the optimal threshold level used in the EFA show that 70 $\pm$ 9% of the detected particles carry viral glycoproteins (red bar) and 43 $\pm$ 2% VP5 (green bar). VP5 is only accessible after permeabilization of the envelope (green and white bar). Formaldehyde fixation of the virions prior to permeabilization didn't influence the staining efficiency, thus both fixed and non-fixed samples are considered for VP5 staining. Results of at least two independent experiments (grey dots). Error bar indicates the standard error of the mean. **(d)** Plot displays the results of the threshold scouting performed to find the optimal threshold level for the EFA experiments. Double stained particles were analysed to calculate the number of detected particles (blue empty circles) and the percentage of double-stained particles (dark red circles) as a function of the detection threshold in the 561 nm channel. The number of particles vs detection threshold obtained for EFA experiments is also plotted after multiplication of the x coordinate by the adjustment factor ($c = 4$). The dotted vertical line indicates the optimal threshold ($T_{\mathrm{col}}^{\mathrm{opt}}$) used in the biophysical studies. Error bars indicate standard deviation from at least 8 images.

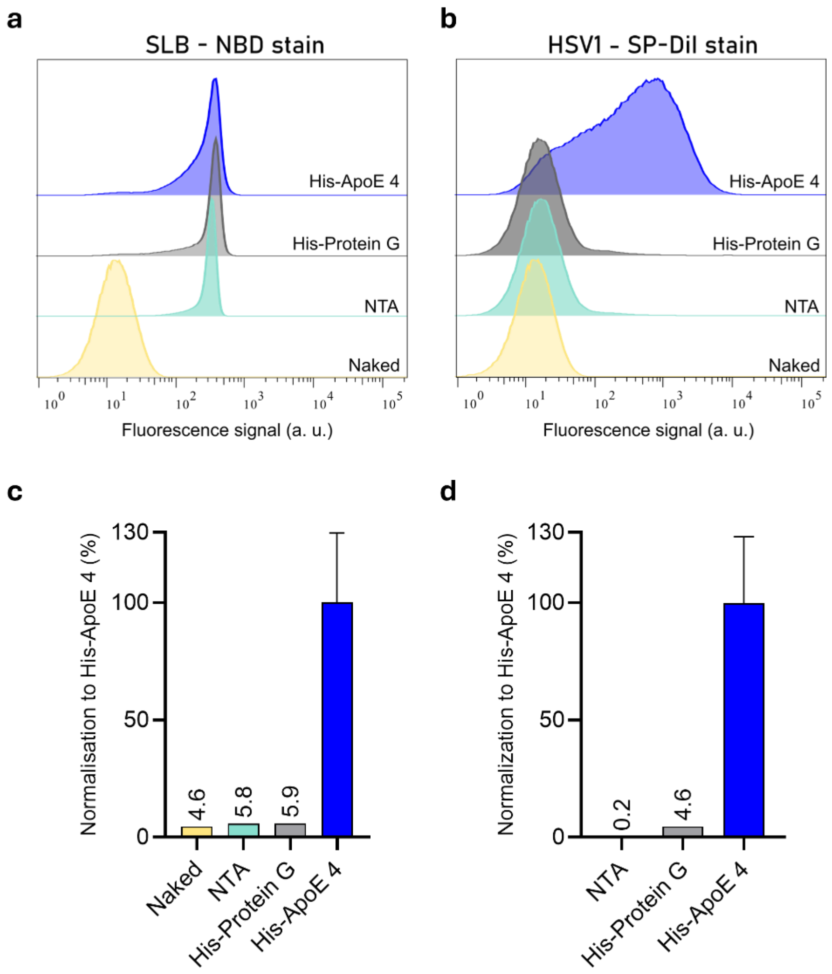

Supplementary Figure 6: HSV1 specifically binds to surface immobilised ApoE 4. POPC:DGS-NTA SLBs containing fluorescent NBD-conjugated lipids were formed onto silica beads and used to immobilise ApoE 4 or Protein G. The beads were then incubated with SP-DiI-stained HSV1. **(a)** Representative flow cytometry histograms demonstrating the presence of a fluorescent bilayer on silica beads in all samples compared to naked beads (Naked). **(b)** Representative flow cytometry histograms showing that SP-DiI-stained virions bind exclusively to surface-bound His-ApoE 4, but not to His-Protein G or NTA-SLB (NTA). The signal obtained from glass beads not incubated with the virus (Naked) is also shown. All histograms are normalised to the value of the mode of each sample. **(c)** Median SP-DiI fluorescent signal from the conditions listed in (b). **(d)** qPCR quantification of the number of HSV1 genome copies from the samples in (c). Data in (c) and (d) are normalised by the average of the His-ApoE 4 signal. Two samples analysed for His-ApoE 4 and one for other conditions.

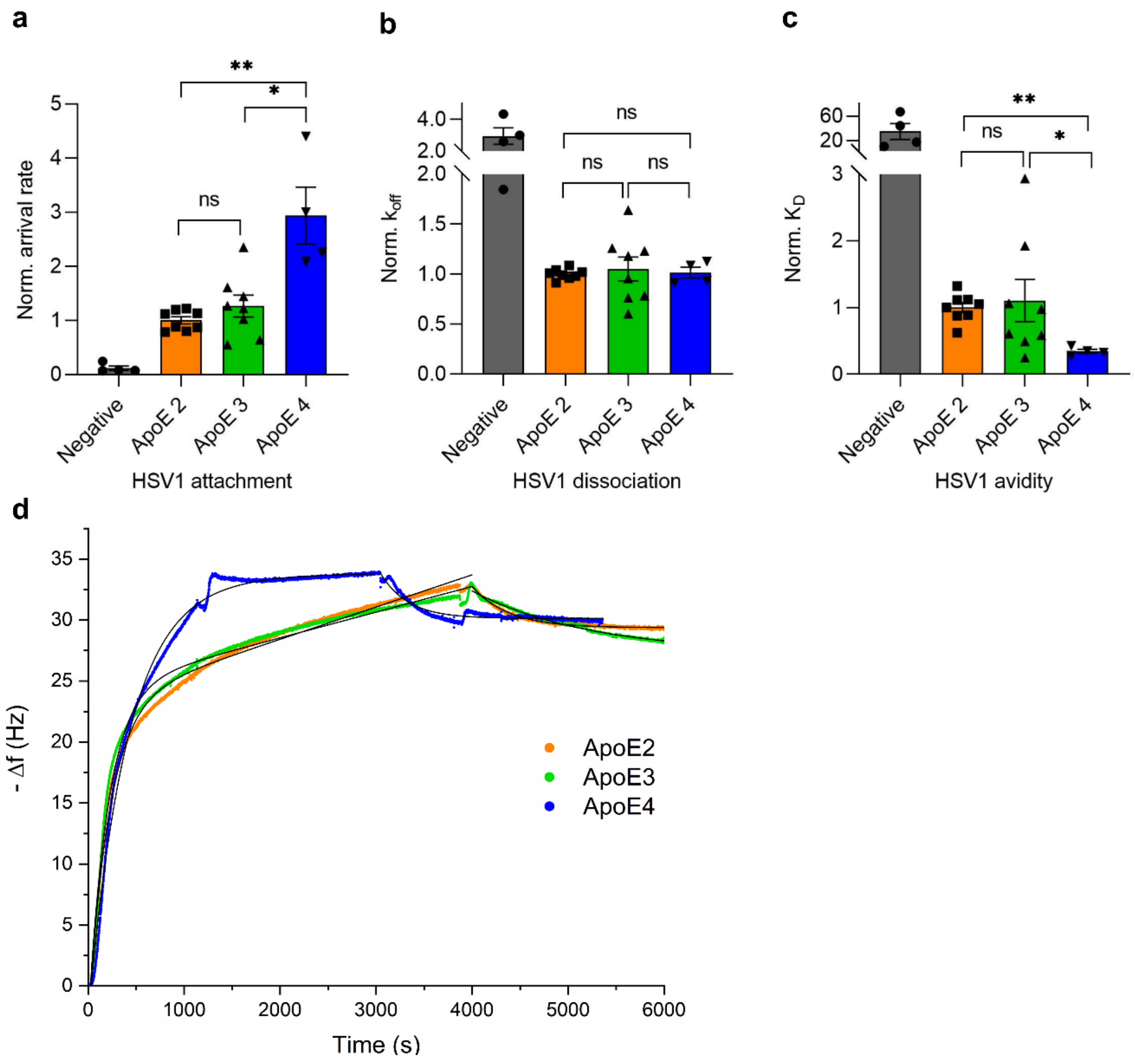

Supplementary Figure 7: **Kinetic analysis (association rate (a), dissociation rate constant (b) and dissociation constant (c)) of the bond formed between HSV1 and surface-immobilised ApoE isoforms using our TIRFM-based equilibrium fluctuation analysis assay.** His-tagged ApoE 2, 3 and 4 were immobilized on a POPC:DGS-NTA membrane surface as described for Fig 3E in the main text. Data were normalised by the average value of ApoE 2 for each experimental repeat. Statistical significance determined using Mann-Whitney test between each pair of isoforms. ns: no significance difference detected, *: $p<0.05$, **: $p<0.005$. A substrate lacking ApoE was used as a negative control. The negative control exhibits a significant difference to all other samples in all graphs (not shown to increase readability). **(d)** Binding of His-tagged ApoE isoforms on a POPC:DGS-NTA bilayer was monitored using QCM-D. All ApoE isoforms were diluted to a concentration of 100 µg/ml in HBS and flown over the bilayer. Protein attachment is detected as a proportional shift of the resonance frequency of the sensor. After saturation, the surface was rinsed in HBS to ensure stable immobilisation. Binding curves were fitted with the function $-\Delta f = A\left[1-\exp\left(-\frac{t-t_0}{\tau}\right)\right]+B\cdot t$ and the detachment with $-\Delta f = C\left[1-\exp\left(-\frac{t-t_0}{\tau}\right)\right]+D$, where A, B, C, D, t_0, τ are fitting parameters. All isoforms show comparable levels of bound proteins after rinsing (ApoE 2: 29 Hz, ApoE 3: 28 Hz, ApoE 4: 30 Hz).

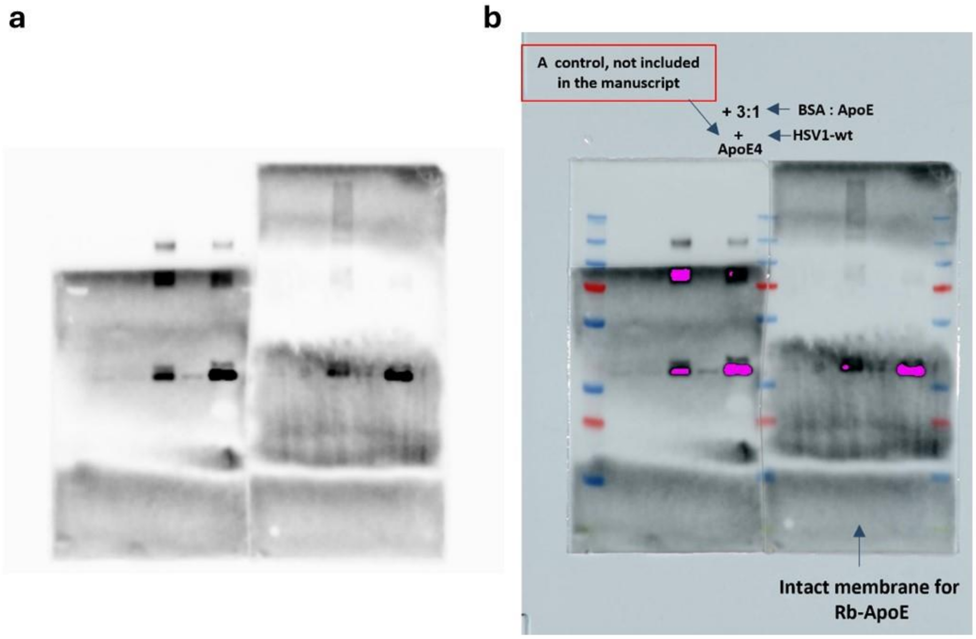

Supplementary Figure 8: Original blots for **Fig. 3B** in the main text without **(a)** or with **(b)** a protein ladder. A control with BSA was included but not presented in the main text.

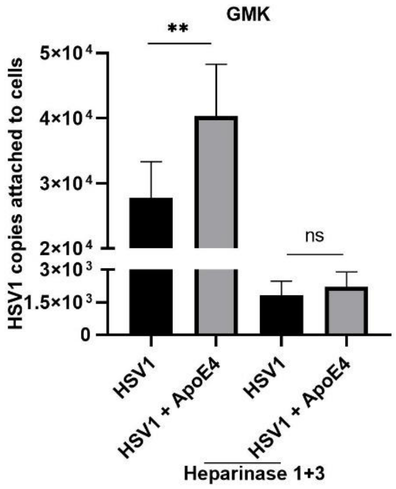

Supplementary Figure 9: The attachment of HSV1 and HSV1+ApoE4 to cells in the absence of heparan sulfate. HSV1 and HSV1+ApoE4 were labelled and purified as in Fig. 3D, 3E, and 4 (described in the Methods section of the main text). The prepared viruses were quantified by qPCR and same amount of HSV1 or HSV1+ApoE4 (50,000 copies) were added to cells for attachment on ice. Prior to adding the viruses, GMK cells were treated with or without heparinase 1 and 3 (1 unit/mL) for 1 h at 37 °C, followed by 3 times PBS wash to remove HS fragments. Virus attachment to GMK was then carried out on ice for 45 min as done in Fig.

4. The results represent two experimental repeats with sub-duplicates in each experiment. Student t-test, significance **: p≤ 0.01. Error bars: mean ± SD.

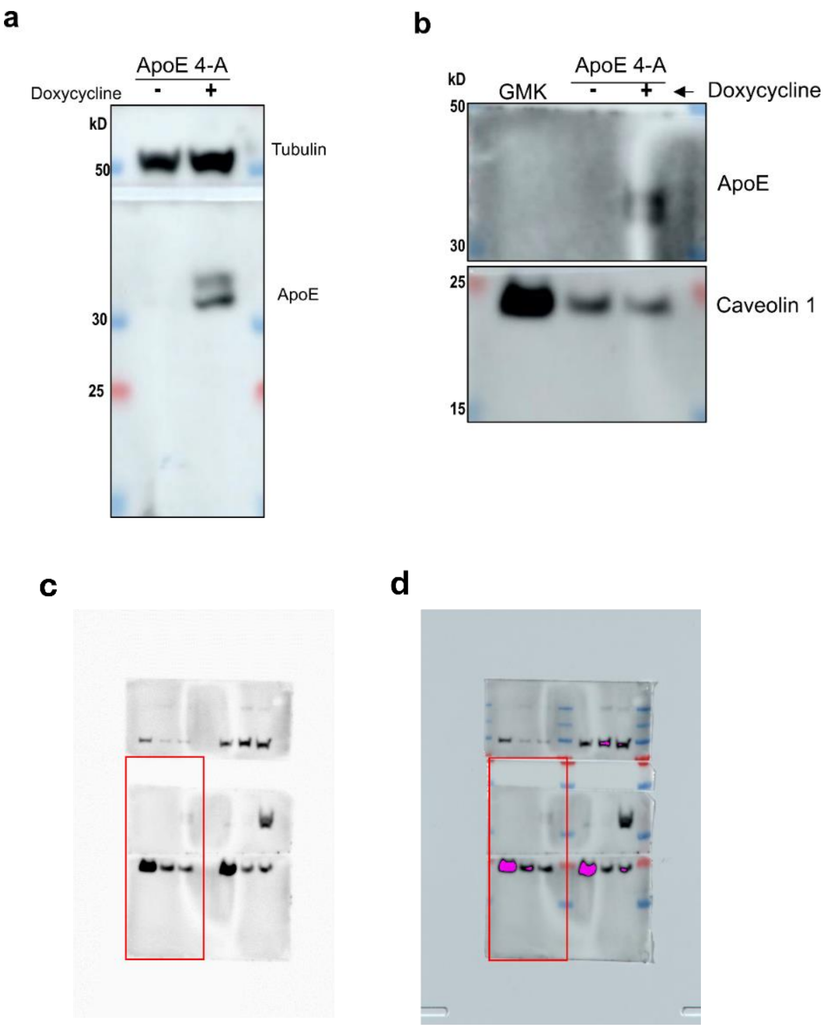

Supplementary Figure 10: Plasma membrane association of ApoE 4 in an ApoE 4 inducible cell line. (a) Induction of ApoE 4 by doxycycline. ApoE 4 inducible cells were prepared in large culture for membrane extraction. The expression of ApoE 4 was induced by adding doxycycline (1 µg/mL) overnight (about 16 h), before the cells were harvested for membrane extraction. Aliquots of both mock-treated and induced cells were collected and analysed by western blot for ApoE expression. Tubulin was probed as a loading control. **(b)** Plasma membrane association of ApoE 4. The presence of ApoE 4 and caveolin 1 were verified by western blot in the extracted and purified plasma membrane samples. Original blots (in the red box) for the blot presented in (b), (**c**) without or (**d**) with a protein ladder. Figure (a) was presented as the original blot.

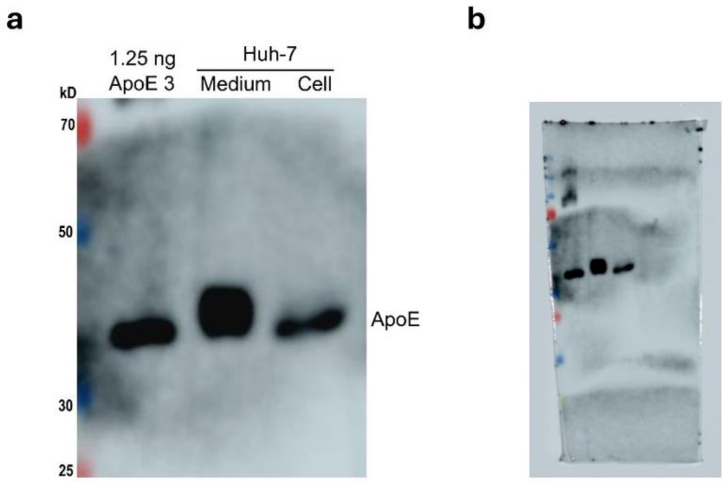

Supplementary Figure 11: ApoE levels in the medium (secreted) and the cells of Huh-7. Huh-7 cells were cultured in a T75 flask with DMEM (10% FBS, 20 mM HEPES, penicillin (0.5 unit/mL) and streptomycin (50 µg/mL)). When close to 100% confluence, the culture medium and cells were quantified and collected for lysates preparation 2and western blot analysis, as described in the methods of the main text. The culture medium was 8 mL and cell counts were 3.5 million. 50 µl of medium sample (1/6 was loaded) and 1 million cells (1/48 was loaded) were lysed and prepared for western blot. Purified ApoE 3 (1.25 ng) was included as a control. The concentration of ApoE in the medium was calculated as 176.574 µg/mL (170 µg/mL = 5 µM) and in cells as 40.43 ng per million cells. Calculations were done based on the intensities of the corresponding bands. (**a**) processed and (**b**) original blots.

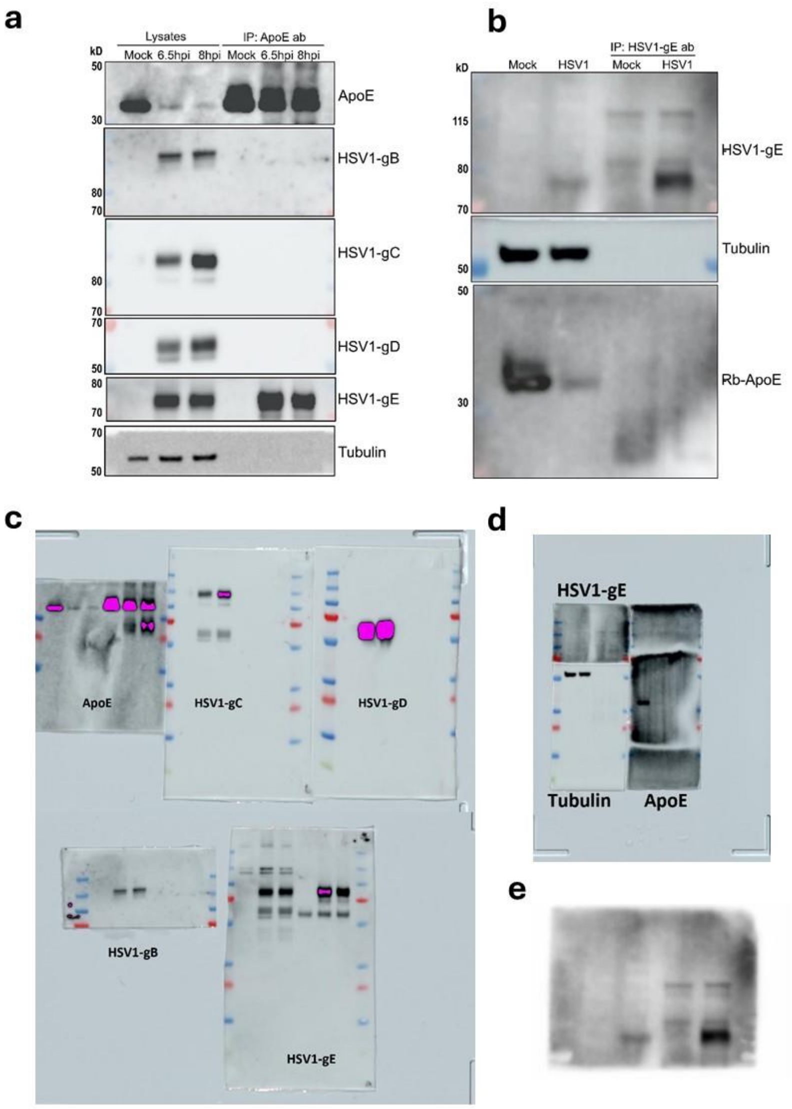

Supplementary Figure 12: Interactions analysis between ApoE and HSV1 glycoproteins. Huh-7 cells (expressing ApoE 3) were mock-treated or infected with HSV1 (MOI 10). Cells were lysed at indicated time points, followed by immunoprecipitation with the antibody against ApoE **(a)** or HSV1-gE **(b)** as described in the methods of the supplementary information. Protein complexes pulled down were separated by SDS page and the indicated proteins were probed by western blot. **(c and d)** Representative original blots for (a). **(e)** Original blots for (b). **(f)** A better exposed blot for HSV1-gE. Tubulin blot was presented as original in (a).

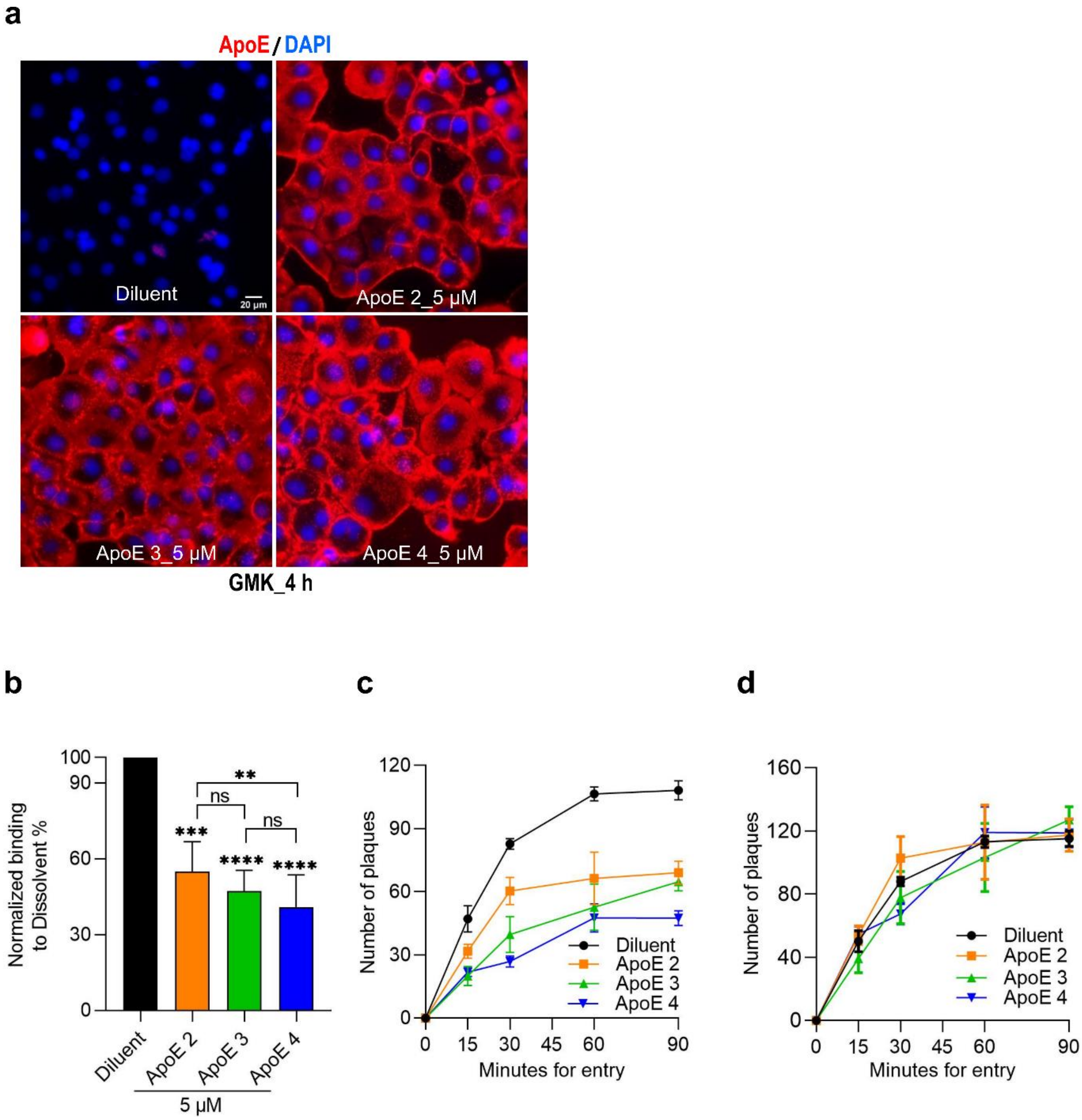

Supplementary Figure 13: (a) ApoE distribution in GMK cells. Diluent or 5 µM ApoE 2, 3, or 4 was diluted in normal culture medium and added to GMK cells in a 24-well plate. After 4 h incubation, cells were fixed with 4% formaldehyde for 15 min and permeabilized with the permeabilization buffer (0.5% Triton X-100, 20 mM glycine in PBS) for 15 min. The fixed samples were incubated with a blocking buffer for 30 min, followed by incubation of primary antibody against ApoE (PA5-27088 ThermoFisher Scientific, 1:1000 in blocking buffer) for 1 h and secondary antibody (A32733 ThermoFisher Scientific, Alexa Fluor^{TM} Plus 647, 1:500) for 1 h. The nuclei were stained with DAPI together with the secondary antibody incubation. All the procedures were carried out at room temperature. Images were taken with Nikon (Japan) Eclipse Ti-E2 microscope, 60X oil objective. Representative images were shown for each group. **(b, c, and d) HSV1 binding, but not entry, is affected by ApoE when added prior to infection. (b)** HSV1 attached to GMK cells, pre-treated with ApoE or diluent, were quantified by qPCR after 1 h binding synchronization on ice. The data of ApoE groups were normalized to the diluent group. The percentages of ApoE groups are 55% (ApoE 2), 47% (ApoE 3), and 42% (ApoE 4). **(c)** HSV1 entry to GMK treated with ApoE or diluent at selected timepoints was quantified by plaque

formation. The average value of the diluent at 90 min was seen as the plateau, to which the rest of the data were normalized. No statistical analysis was performed. **(d)** HSV1 entry efficiencies under different conditions were calculated by dividing the number of plaques by the binding ratios as shown in (b), followed by normalization as done for (c). The normalized entry efficiencies of each ApoE group were compared to the diluent group individually at the same time points. No significance was observed from any of the statistical analysis for (d). Results represent three or more independent repeats. Student t-test, ***: p≤0.001, and ****: p≤0.0001. Error bars: mean ± SD.

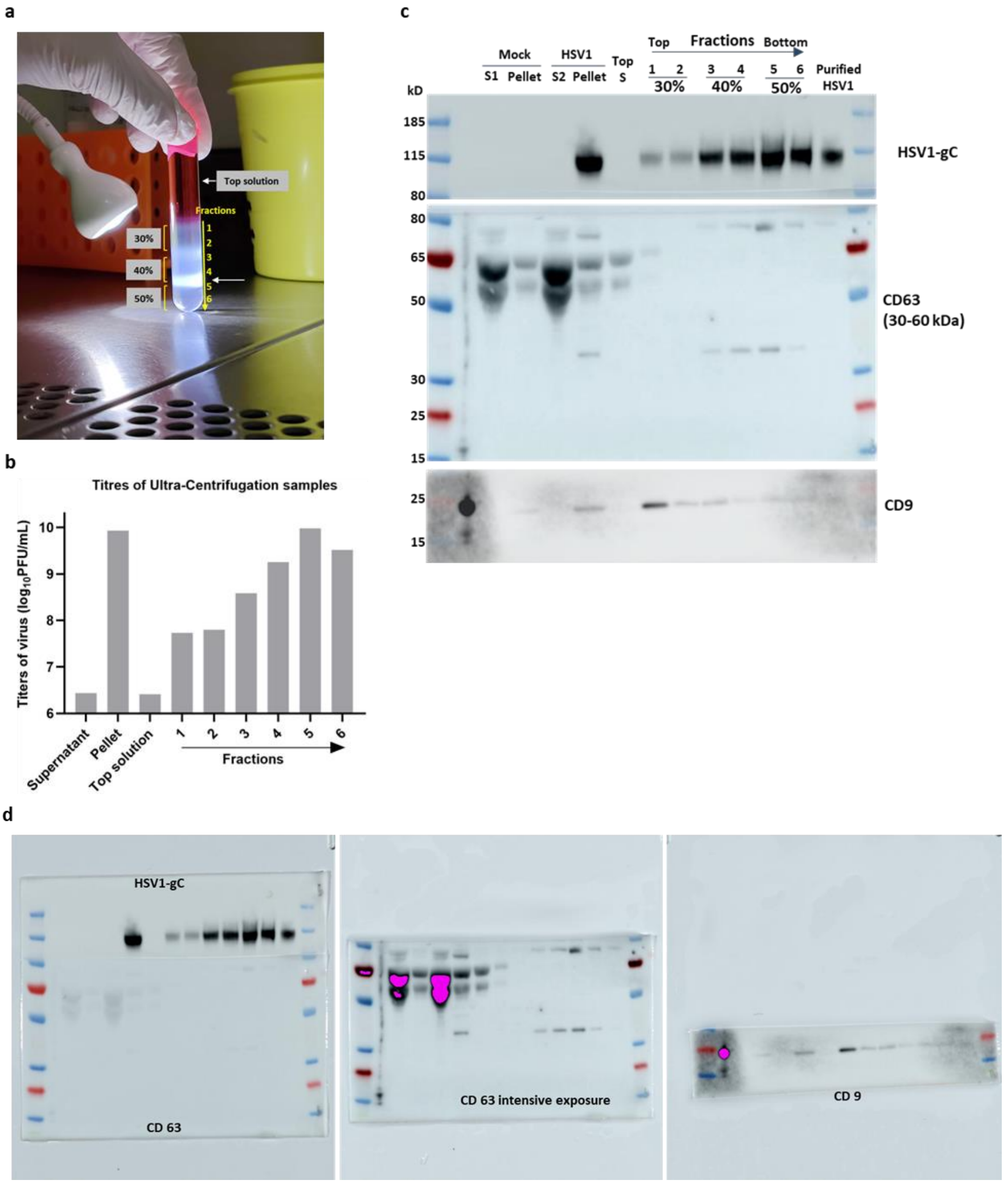

Supplementary Figure 14: The distributions of EVs after sucrose-based preparation of HSV1 stock. (a) A picture of the samples after ultracentrifugation through sucrose gradients. Top solution (Top S) was collected

as an individual fraction. Samples in sucrose gradients were fractioned as 1 mL/fraction. **(b)** Plaque assay of the fraction samples as collected in (a) and samples aliquoted from different stages of the protocol. Supernatant: HSV1 infected GMK medium after pelleting HSV1 with high-speed centrifugation at 18,000 rpm, 2h. Pellet: virus pellets were resuspended in infection medium (DMEM, 1% FBS). **(c)** Mock treated and HSV1 infected GMK samples (as in **a** and **b**) were probed with the indicated markers. S1/S2: supernatant of mock or HSV1 infected GMK medium after pelleting HSV1. Pellet: resuspended samples of the mock pellet or virus pellet. (d) original blots for (c).

Supplementary Table 1: Summary of the fitted (A_1, A_2 and y_0) and derived (k_{off}1 and k_{off}^2) parameters from the double exponential fits of the normalized dissociation curves for the data presented in figures 5, 6, and 7. Each dissociation curve was normalized so that the total number of particles is expressed as a fraction of 1 and then fitted with a double exponential function: $f(t) = A_1 \cdot \exp(-{k_{\mathrm{off}}}^1 \cdot t) + A_2 \cdot \exp(-{k_{\mathrm{off}}}^2 \cdot t) + y_0)$. All values are given as mean ± SD (standard deviation) from 3 independent experiments.

Figure #	Probed surfaces	Fluorescently labelled particles	Fitted parameters			Derived parameters	
			A_1	A_2	y_0	k_{off}^1 (s^{-1})	k_{off}^2 (s^{-1})
5	nSLBs	HSV1	0.24 ± 0.09	0.077 ± 0.040	0.841 ± 0.054	0.0230 ± 0.0064	0.00146 ± 0.00067
		HSV1+ApoE 4	0.40 ± 0.25	0.121 ± 0.031	0.773 ± 0.082	0.0268 ± 0.0057	0.00207 ± 0.00075
6	HS films	HSV1	0.52 ± 0.05	0.183 ± 0.078	0.620 ± 0.071	0.0768 ± 0.0099	0.00816 ± 0.0008
		HSV1+ApoE 4	0.64 ± 0.06	0.281 ± 0.111	0.452 ± 0.133	0.0709 ± 0.0217	0.00706 ± 0.00099
7	HEK-†	HSV1	0.47 ± 0.11	0.148 ± 0.020	0.658 ± 0.063	0.0100 ± 0.0016	0.00080 ± 0.00014
	HEK+†		0.40 ± 0.05	0.145 ± 0.027	0.708 ± 0.055	0.0111 ± 0.0046	0.00110 ± 0.00041

† HEK- and HEK+ represent the nSLBs from HEK cells without and with the induction of ApoE 4 expression, respectively.
